# Neuronal Necroptosis Drives Neuroinflammation and Cognitive Decline Independent of Neuronal Cell Death

**DOI:** 10.14336/AD.2025.0805

**Published:** 2026-09-16

**Authors:** Nidheesh Thadathil, Nicholas A Wolf, Roman Wolf, Carlos Manlio Díaz-García, Sreemathi Logan, Daniel B Owen, Kevin Pham, Willard M Freeman, Arlan Richardson

**Affiliations:** ^1^Department of Biochemistry and Physiology, University of Oklahoma Health Sciences Center, Oklahoma, OK 73104, USA.; ^2^Center for Geroscience and Healthy Brain Aging, University of Oklahoma Health Sciences Center, Oklahoma City, OK, 73104, USA.; ^3^Oklahoma City VA Medical Center, Oklahoma, OK 73104, USA.; ^4^Oklahoma Medical Research Foundation, Oklahoma, OK 73104, USA.

**Keywords:** Necroptosis, brain, neuroinflammation, cognition, neurodegeneration

## Abstract

Markers of necroptosis increase in neurons with neurodegenerative diseases and aging. Using a novel knockin mouse model that overexpresses the terminal necroptosis effector gene MLKL specifically in neurons (nMlkl-KI), we studied the impact of inducing necroptosis in neurons of young/adult mice on cognition. At 6-months of age, nMlkl-KI mice exhibited a 7-fold and 3-fold increase in MLKL monomer expression in the cortex and hippocampus, respectively. Correspondingly, MLKL-oligomer levels increased 3- to 4-fold in these brain regions, indicating necroptosis activation. The increased necroptosis was associated with an induction of neuroinflammation as shown by an increase in transcript levels of inflammatory markers and increased Iba-1 expression in the cortex and hippocampus. At 12-months of age, nMlkl-KI mice exhibited significant cognitive impairment compared to control mice as measured by a continuous home-cage discrimination learning with the Noldus PhenoTyper. For example, cumulative learning index during the reversal phase and cognitive flexibility were dramatically reduced in nMlkl-KI mice as compared to the control mice. Unbiased stereological analysis revealed no loss in neuronal number in the cortex and hippocampus, suggesting neuronal dysfunction rather than neuronal death was responsible for the reduced cognition observed in the nMlkl-KI mice. Transcriptomic analysis of the cortex revealed an upregulation of pathways associated with age-related neurodegenerative diseases (e.g., Parkinson’s, Alzheimer’s, Huntington’s) as well as chemokine and TNF signaling in the nMlkl-KI mice. In contrast, the neuroactive ligand-receptor interaction pathway was downregulated. Collectively, these data show for the first time that the overexpression of MLKL in neurons leads to a loss in cognition in the absence of neuronal cell death, demonstrating that increased MLKL can interfere with neuronal functions involved in cognition.

## INTRODUCTION

Aging is closely associated with chronic inflammation, often referred to as "inflammaging," which is characterized by persistent low-grade immune activation that contributes to tissue damage, metabolic dysfunction, and age-related diseases such as cardiovascular disorders, neurodegeneration, and cancer [1, 2]. Although it is recognized that inflammaging plays an important role in aging and most age-related diseases, it is unclear what pathways are responsible for inflammaging. Necroptosis is a regulated form of necrosis that triggers inflammation and is mediated primarily by receptor-interacting serine/threonine-protein kinase 1 (RIPK1), RIPK3, and mixed lineage kinase domain-like protein (MLKL) [3]. Unlike apoptosis, which is typically immunologically silent, necroptosis leads to the release of damage-associated molecular patterns (DAMPs) from cells that trigger a robust inflammatory response [4]. Our group was the first to show that necroptosis increased with age in mice (e.g., adipose tissue [5], liver [6], and brain [7]). We also showed that necroptosis was reduced in adipose tissue by dietary restriction [5] and in long-lived Ames dwarf mice [8] and increased in the liver of *Sod1^-/-^* mice [8, 9], which is a model of accelerated aging and frailty [10]. Importantly, the age-related increase in necroptosis was associated with an increase in inflammation, and inhibiting necroptosis reduced inflammation [9, 11].

While our group was the first to report that necroptosis increased with “normal” brain aging [7], necroptosis had been reported to be associated with various neurodegenerative diseases, e.g., Alzheimer’s disease [12-14], Parkinson’s disease [15-17], multiple sclerosis [18], Huntington disease [19], and amyotrophic lateral sclerosis [20]. We observed a region-specific increase in markers of necroptosis in the brains of old mice that occurred primarily in the neurons of the cortex and hippocampus and was associated with increased neuroinflammation [7]. While neuronal loss is a hallmark of neurodegenerative diseases such as Alzheimer's [21] and Parkinson's [22], neuronal cell loss during “normal” brain aging appears to be minimal [23-25]. This raised the possibility that necroptosis could impact the function of the aging brain through a mechanism other than neuronal cell loss. In a recent study, we showed that overexpressing MLKL in neuronal cells in the absence of cell death led to both Ca^2+^ dyshomeostasis and metabolic remodeling [26], demonstrating for the first time that MLKL might negatively impact neuronal function.

The goal of this study was to determine the *in vivo* impact of specifically inducing necroptosis in neurons on brain function. Using knockin mice that overexpress MLKL specifically in neurons, we show that increased necroptosis in the cortex and hippocampus was associated with an increase in the expression of markers of neuroinflammation and reduced cognition, which occurred in the absence of neuronal cell loss. These data show for the first time that increased neuronal necroptosis can lead to reduction in brain function *in vivo* in the absence of neuronal cell loss.

## MATERIAL AND METHODS

### Animals

Mlkl knockin (KI) mice were generated in the C57BL/6J mice background by ViewSolid Biotech Inc. (Oklahoma City, OK) with a transgene construct containing the Mlkl-cDNA, which had a 3xFlag (flag)-tag sequence on the 3′-end of the cDNA and a stop-cassette flanked by loxP sites inserted at the 5′ end of the transgene, that was inserted into mouse Rosa26 locus [27]. When the stop-cassette is removed, the Mlkl-transgene is expressed under the control of the endogenous Rosa26 promoter. Hemizygous Mlkl-KI female mice were crossed to male homozygous Slick-H-Cre (Thy1-cre/ERT2,-EYFP)HGfng/PyngJ) transgenic mice obtained from The Jackson laboratory (Bar Harbor, Main, Strain #:012708) to produce mice that express MLKL specifically in neurons when treated with tamoxifen, i.e., nMlkl-KI mice. Tamoxifen (Millipore Sigma; catalog number# T5648) was dissolved in corn oil and at 45-days of age the mice were administered tamoxifen (60 mg/kg body weight) or corn oil by intraperitoneal injection for 5 consecutive days. Corn oil injected control mice were housed in separate cages to avoid carryover of tamoxifen.

Three-, 6-, and 12-months-old male mice were used in these experiments. The mice were group-housed in ventilated cages at 20 ± 2 °C, on a 12-h/12-h dark/light cycle, and fed a laboratory rodent chow (5053 Pico Lab, Purina Mills, Richmond, IN) *ad libitum*. At specific ages, mice were anesthetized with isoflurane using anesthesia machine (Kent Scientific Somnosuite, Torrington, CT 06790, USA) at flow rate of 500 mL/h for induction and 50 mL/h for maintenance. Anesthetized mice were perfused with phosphate-buffered saline (1X PBS, pH 7.4), and the brain was carefully dissected. The brain was separated into two hemispheres, and one hemisphere was transferred to 4% paraformaldehyde (PFA) for fixation for 24 h followed by 30% sucrose (w/v) solution at 4 °C for 48 h. From the other hemisphere, brain regions such as cortex, hippocampus, brain stem, hypothalamus and cerebellum were collected, snap frozen in liquid nitrogen, and stored at -80 °C until analyzed. The mice were generated and maintained in the animal facility at the Oklahoma City Veterans Affairs Health Care System Animal Facility, and all procedures were approved by the Institutional Animal Care and Use committee at the Oklahoma City Veterans Affairs Health Care System Animal Facility.

### Cognitive analysis

To assess the effect of MLKL overexpression in neurons on cognition, we performed cognitive analysis using the Noldus PhenoTyper as described by Logan et al. [28, 29]. This non-invasive system measures cognition by a food-reward based spatial memory task divided into two learning phases: an initial discrimination learning phase and a reversal learning phase. The Phenotyper system also allows the investigators to measure movement parameters and has been shown to detect deficits in cognition arising from aging [28] or oxidative stress [29, 30]. In the behavioral analysis, untreated young C57BL/6J (4-6 months old) mice were also included with tamoxifen treated and corn oil treated Slick H Cre - Rosa Mlkl mice at 12 - 13 months of age. At the beginning of the study, animals were placed individually in the PhenoTyper Model 3000, Noldus Information Technology, Netherlands) for a 6-h adaptation period. The activity of the animals was continuously recorded for 90 h of light/dark cycle. An infrared-sensitive video camera above the arena recorded all movements using the X-Y coordinates of the center of gravity of each animal. These data were sampled at a rate of 15 frames per second and smoothed using EthoVision software (EthoVision XT 11.5, Noldus Information Technology, Wageningen, The Netherlands) as previously described [31, 32]. Immediately prior to initiation of video tracking and activation of the task protocol, a food pellet dispenser is connected and a cognition wall (Noldus Information Technology, Netherlands) placed into the Phenotyper, consisting of an insert with three entrances placed in front of the spout of a food dispenser, as described in detail in previous publications [33, 34]. Body weights were taken at the beginning and at the end of the study. For the initial discrimination phase of the study, the animal was required to pass through the left entrance of the cognition wall to obtain a food reward (Dustless Precision Rodent Pellets, F05684, Bio-Serv, Flemington, NJ), which were dispensed using a Fixed Ratio (FR) 5 schedule throughout the testing period. After 48 h of discrimination learning, the correct response was changed to the right entry requiring the animal to extinguish the previous learning and acquire a new response. Success rates for both initial discrimination and reversal learning were calculated post hoc and defined as the percentage of correct entries of the trailing 30 entries through any of the entrances of the cognition wall. The percentage of animals in each age group reaching success rates was calculated. The following dependent variables to reach a specific success rate (80%) were calculated for both initial discrimination and reversal learning: percent of animals reaching criterion, entries to criterion, errors to criterion and time (hour) to criterion.

### Immunofluorescence staining

Immunofluorescence staining was performed as described by Thadathil et al. [7]. Briefly, 30-μm serial coronal brain sections were sectioned on a cryostat (Leica CM 3050 S, Deer Park, IL). Sections were rinsed twice with PBS (pH 7.4) followed by permeabilization with 0.25% triton X-100 in PBS for 10 min. After washing with PBS, sections were blocked with 2.5% bovine serum albumin containing 5% donkey or goat serum (Abcam, Waltham, MA) for 1 h at room temperature. Sections were incubated overnight at 4 °C with primary antibodies prepared in blocking buffer against Flag (1:250, Cat. No. SAB4301135-50UG, Sigma-Aldrich, St. Louis, MO), neuronal nuclear antigen (NeuN, 1:800, Abcam, Cat. No. ab104224), ionized calcium binding adaptor molecule 1 (Iba-1, 1:100, Cat. No. ab48004, Abcam, Waltham, MA), or glial fibrillary acidic protein (GFAP, 1:300, Cat. No. 3670, Cell Signaling Technology, Danvers, MA). After washing the sections with PBS five times, fluorescent labelled secondary antibodies in blocking buffer were added, either alone or in combination, based on the primary antibody, and incubated overnight at 4°C. The following secondary antibodies were used: Donkey anti-rabbit Alexa Fluor 647 (Cat. No. A-21244), Goat anti-mouse Alexa Fluor 514 (Cat. No. A-31555) from Thermofisher Scientific, Dallas, TX, and Goat anti-mouse Alexa Fluor 594 (Abcam, Cat. No. ab150120). Stained sections were then washed with PBS for 5 times and mounted using ProLong™ Diamond antifade mountant (Thermofisher Scientific, P36961) which provided labeling of all cell nuclei with DAPI. For the co-localization of flag (transgene) with brain cell types (neuron, microglia, and astrocytes), serial coronal sections were taken, and the number of cells positive for the different antibodies (NeuN, Iba-1 and GFAP) was counted in similar areas (dentate gyrus, CA1 and CA3 hippocampus and cortex) in consecutive serial sections. The Slick-H Cre mice show strong yellow fluorescent protein (YFP) labeling in projection neurons across many brain regions as expected for the *Thy1.2* promoter; however, the intensity of YFP is less in other neuronal cell types [35]. Therefore, neurons were co-labelled with NeuN (in green) unless otherwise mentioned. All the imaging was conducted with a confocal microscope (Zeiss LSM 710) at 200X and 630X magnifications in five non-overlapping fields per mouse and then averaged by an investigator blinded to the groups. We primarily focused on layers V and VI of the cerebral cortex based on previously reported region-specific activation of necroptosis in the aging brain [7]. Four to six mice per age group were used each staining, and images were quantified using ImageJ software (U.S. National Institutes of Health, Bethesda, MD, USA) and the total number of cells stained with DAPI.

### Western blotting

Fifteen mg of tissue from the cortex or hippocampus were homogenized in RIPA lysis buffer (ThermoFisher Scientific, Waltham, MA) containing phosphatase and protease inhibitor cocktail (Goldbio, St Louis MO, Cat. No. GB-108-2). Western blotting was performed using 30 μg protein/well as previously described [7, 36]. Primary antibodies against the following proteins were used: MLKL (Millipore, Cat. No. MABC604), Flag (Cat. No. F71804, Sigma-Aldrich, St. Louis, MO), β-tubulin (Cat. No. T5201), and GAPDH (Cat. No. G8795) were purchased from Sigma-Aldrich (St. Louis, MO). HRP-linked anti-rabbit IgG, HRP-linked anti-mouse IgG, or HRP-linked anti-rat IgG were used as secondary antibodies (Cell Signaling Technology, Danvers, MA). Membranes were imaged with ChemiDoc imaging system (Bio-Rad, Fort Worth, TX) and quantified using ImageJ software (U.S. National Institutes of Health, Bethesda, MD, USA).

MLKL oligomers were detected by western blotting under non-reducing conditions as described by Miyata et al. [37]. The hippocampus and cortex were homogenized in RIPA lysis buffer, and protein quantification was performed as described above. Forty μg of protein was resolved under non-reducing conditions without SDS in running buffer and polyacrylamide gel. MLKL oligomers were detected on the gels as larger than 200 kDa and quantified using the antibody to MLKL as described above. Anti-MLKL antibody (Millipore, Cat No. MABC604) was used to detect MLKL-oligomers.

### Quantitative real-time PCR and RNAseq Analysis

Relative mRNA levels of necroptosis and inflammatory markers in mouse cortex were analyzed as described by Thadathil et al. [7]. Briefly, total RNA was extracted using the RNeasy kit (Qiagen, Valencia, CA, USA) from 15 mg frozen cortex. First-strand cDNA was synthesized using a high-capacity cDNA reverse transcription kit (ThermoFisher Scientific Waltham, MA), and quantitative real-time PCR was performed with ABI Prism using Power SYBR Green PCR Master Mix (ThermoFisher Scientific, Waltham, MA). β-actin was used as the endogenous control. The 2^-ΔΔCT^ comparative method was used to quantify the relative changes in gene expression and are presented as fold change.

RNAseq analysis was performed on RNA isolated from brain cortex using NEBNext Ultra II Directional RNA Library Prep Kit for Illumina (New England Biolabs, Ipswich,) as previously described [38]. Paired-end 150 bp read sequencing was performed, in six biological replicates per group, on an Illumina NextSeq 500 sequencing platform. Raw reads, in FASTQC format, were trimmed of adaptor sequences using the Galaxy software. Low-quality bases at the beginning and end of reads were removed, then the quality was assessed using FastQC. Trimmed quality reads were aligned to the *Mus musculus* genome reference (GRCm39/mm39) using STAR v2.4.0h. Gene-level read counts were determined using HTSeq v0.5.3p9 with the GENCODE Release M29 (GRCm39) annotation. Read-count normalization and differential expression analyses were performed using DESeq2. Differentially expressed genes were organized in similar phenotype variation patterns observed in the control and nMlkl-KI mice. Functional Enrichment analysis was performed using specialized packages from Express Analyst to identify enriched Kyoto Encyclopedia of Genes and Genomes (KEGG) pathways among the differentially expressed genes [39].

### Neuronal density/cell count analysis

Blinded manual neuron counts in hippocampal and neocortical regions (frontal, parietal, temporal, and occipital cortices) was performed by unbiased, stereology by SRC Biosciences (Tampa, FL) as described previously Mouton et al. [40, 41] using paraformaldehyde fixed hemibrains (n=3/group) in sucrose. SRC Biosciences used unbiased systematic random sampling, stereological probes and other procedures to exclude all known sources of methodological bias [40, 41]. These stereological analyses allowed the quantification of NeuN-immuno-stained with cresyl violet counterstain in five anatomically distinct regions of interests. All stereology studies were carried out by trained technicians and supervisors blind to the treatment groups. Neuron counts were done using a commercial integrated hardware-software stereology system (Stereologer, SRC Biosciences, Tampa, FL). Systematic-random sampling was then used to select a subset uniform, systematically spaced sections through each region of interest (ROI), establishing the section sampling fraction (SSF). At approximately 125-150 x-y sampling sites per ROI, the optical fractionator method was applied to estimate total NeuN-positive neuron number. ROIs were delineated on each section using anatomical landmarks. Tissue volumes were quantified using the Cavalieri-point counting method and total subregion volume calculated according to Mouton et al. [41]. Sampling was done using a 10 µm optical disector height with exclusion planes on the top, bottom and left sides to minimize edge effects. A guard zone of 1-2 µm was applied in the z-axis to avoid surface artifacts. Section thickness was measured at every third or fourth sampling site to estimate the thickness sampling fraction (TSF).

Total neuron number was calculated according to the optical fractionator formula: N = ΣQ- • F1 • F2 • F3 where N is the total estimated neuronal number, ΣQ- is the number of neurons counted; F1 = 1/ SSF = 1/ # sampled sections /total # of sections through reference space; F2 = 1/ area sampling fraction (ASF) = 1/area disector frame/ area of sampling step; F3 = 1/TSF = 1/disector height (um)/ mean post- processing section thickness (um).

### Statistical Analyses

All experiments were performed in multiple independent replicates per group as described. Statistical analyses were performed using GraphPad Prism 10 and JMP v15.2.0 (SAS Inc.) Behavioral data were analyzed using an age × group × time repeated measures ANOVA using JMP as previously reported by Logan et al. [28]. For comparisons involving more than two groups, statistical analysis was performed using two-way ANOVA followed by Tukey post hoc multiple comparison tests. Sample sizes are noted for each experiment with significance denoted as *p < 0.05, **p < 0.01, ***p < 0.001. For analyses involving 6-month-old mice, where sample sizes were small (n = 5 for control and n = 4 for nMlkl-KI), assessment of data normality was not reliable; therefore, group comparisons were performed using the non-parametric Mann-Whitney U test. All statistical tests were two-sided and data presented as mean ± SEM. Differential gene expression analysis was performed using DESeq2, which applies a Wald test on a negative binomial generalized linear model to assess significance.

**Figure 1.**
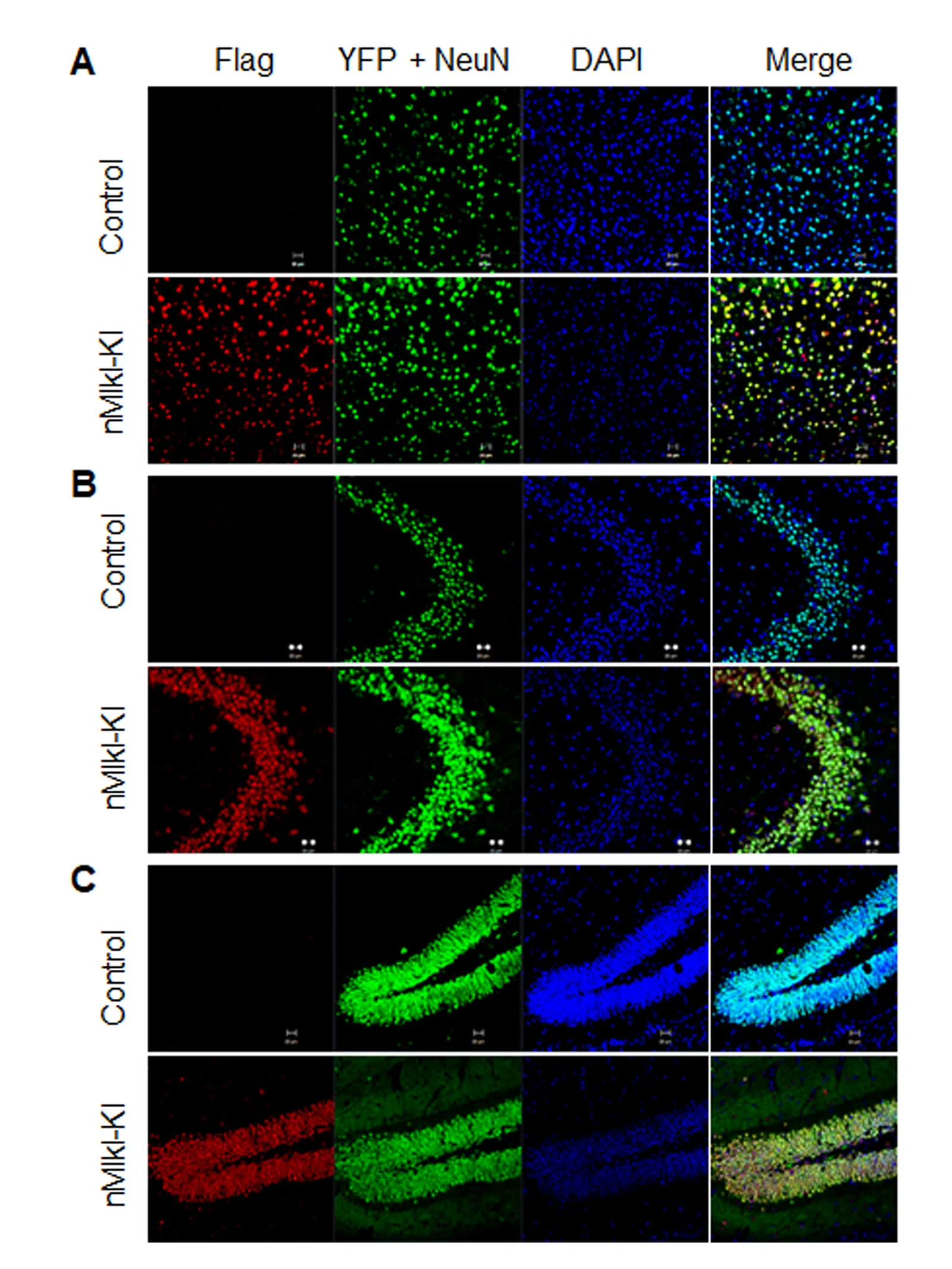
**MLKL expression is specific to neurons in nMlkl-KI mice**. Confocal micrographs show the expression of Flag-tagged MLKL in the cortex layer V (A) and the hippocampus CA3 (B) and dentate gyrus (C) regions of 2.5-month-old control and nMlkl-KI mice. The Flag-tagged Mlkl transgene is shown in red, the neuronal marker NeuN+ yellow fluorescent protein is shown in green, and DAPI is shown in blue. Scale bar: 20 μm.

## RESULTS

### Characterization of nMlkl-KI mice

To generate mice that overexpress MLKL specifically in neurons, we used a novel knockin (Mlkl-KI) mouse model we developed [27] that was crossed into Slick-H Cre transgenic mice, a pan-neuronal Cre under the Thy1 promoter [35]. This leads to the neuron-specific expression of the Mlkl-transgene upon tamoxifen administration, which are designated as nMlkl-KI mice. The characterization of the nMlkl-KI mice is presented in Supplementary Figure 1A, showing that the Mlkl-transgene (identified by the Flag-tag) was expressed in the cortex and hippocampus of the mice treated with tamoxifen and was absent in other organs of mice treated with tamoxifen as well as control mice injected with corn oil. The MLKL transgene was expressed in all brain regions and the spinal cord in tamoxifen injected mice and was absent in control group (Supplementary Fig. 1B). The expression of the Mlkl-transgene varies considerably from one brain region to another with the highest expression occurring in the brain stem and spinal cord. Because our primary interest was on the impact of overexpressing MLKL on cognition, we focused on the cortex and hippocampus in this study.

**Figure 2.**
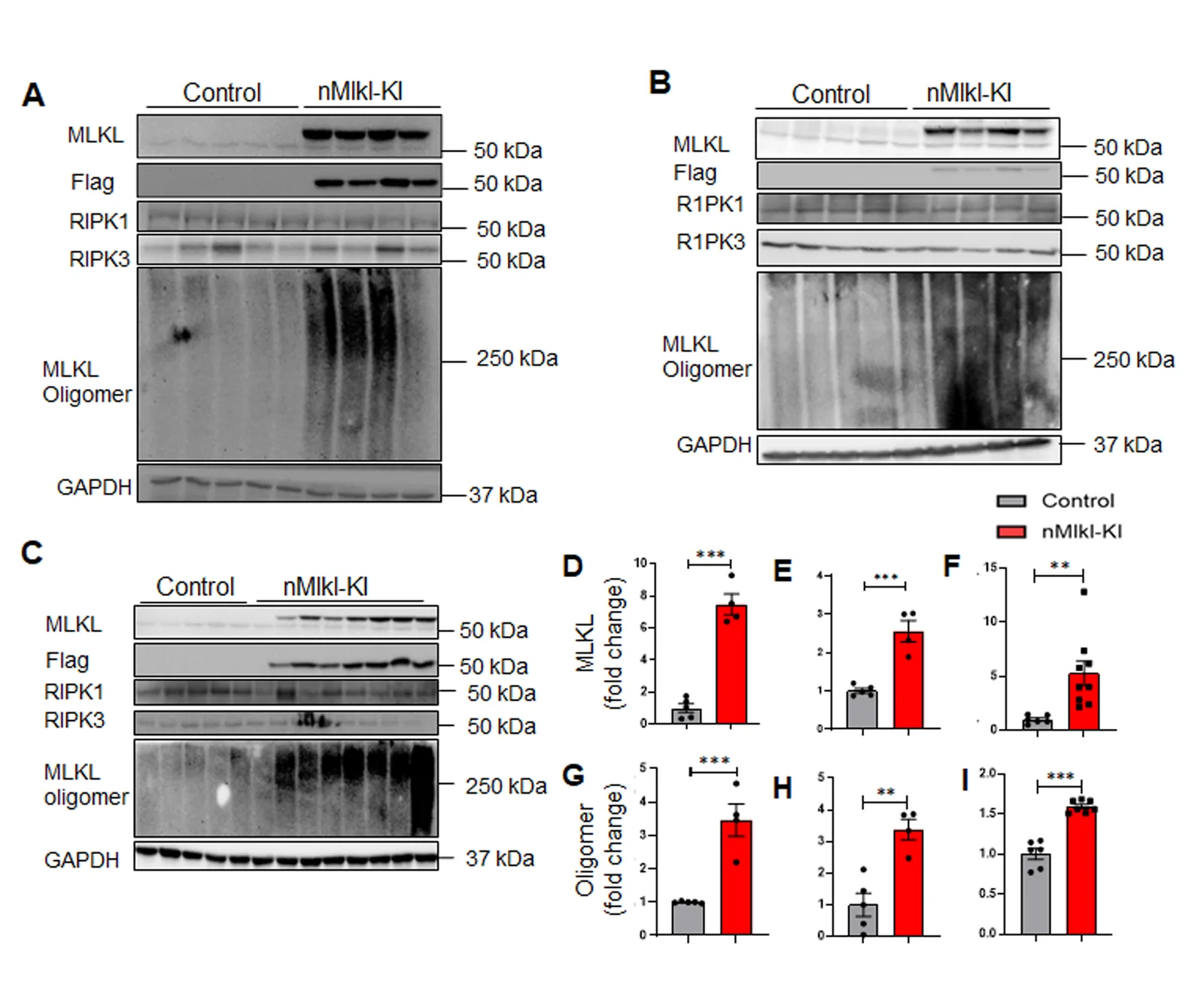
**MLKL overexpression induces necroptosis in nMlkl-KI mice**. The levels of RIPK1, RIPK3, and MLKL monomers and oligomers were measured in brains of 6- and 12-month-old control and nMlkl-KI mice. Levels of the MLKL from the transgene (Flag), MLKL-monomers (endogenous and transgene), RIPK1, RIPK3, and MLKL-oligomers are shown for: 6-month-old cortex (A), 6-month-old hippocampus (B), and 12-month-old cortex (C). The graphs in D through I show the quantification of MLKL levels and oligomerization as fold changes normalized to GAPDH in 6-month-old cortex (D, G), 6-month-old hippocampus (E, H), and 12- to 13-month-old cortex (F, I). Data were obtained from 6-month-old control mice (n = 5), 6-month-old nMlkl-KI mice (n = 4), 12-month-old control mice (n = 6), and 12-month-old nMlkl-KI mice (n = 9) and are expressed as the mean ± SEM. Group comparisons were performed using the non-parametric Mann-Whitney U test. ***p≤ 0.001, **p≤ 0.01.

To confirm that the Mlkl-transgene was expressed specifically in neurons, we used immunofluorescence to identify the cells that were expressing the Flag-tagged transgene. The images in Figure 1 and Supplementary Figure 1C show that the expression of the Mlkl-transgene in the cortex and the CA3 and dentate gyrus regions of the hippocampus were only detected in the nMlkl-KI mice. Co-localization of the Flag-tag with NeuN and YFP confirmed that the Mlkl-transgene was expressed specifically in the neurons of the cortex and hippocampus of the nMlkl-KI mice (Fig. 1 and Supplementary Figure 1C) as would be expected when Mlkl-KI mice crossed to Slick-H Cre transgenic mice are treated with tamoxifen [34].

**Figure 3.**
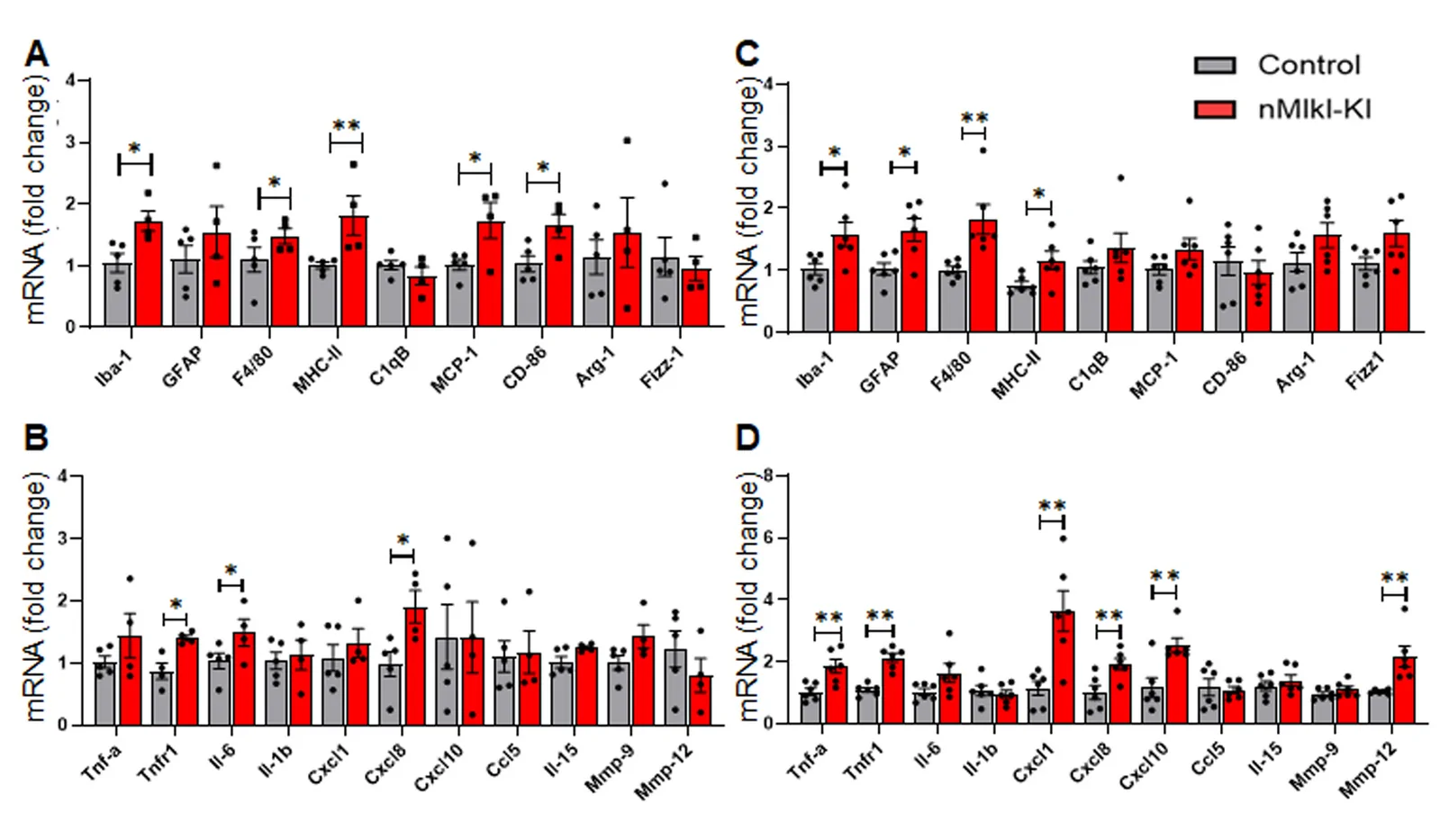
**MLKL over expression results in an increase in markers of inflammation in the cortex of nMlkl-KI mice**. The transcript levels of neuroinflammatory and chemokine genes were measured in the cortex of control and nMlkl-KI mice. The transcript levels of inflammatory markers (Iba-1, GFAP, F4/80, C1qB, MCP-1, CD-86, Arg-1, and Fizz-1) in mice are shown at 6- (A) and 12- (C) months of age. Transcript levels of inflammatory cytokines and chemokines (IL-6, TNF-α, TNFR1, IL-1β, Cxcl1, Cxcl8, Cxcl10, Ccl5, IL-15, Mmp-9 and Mmp-12) are shown at 6- (B) and 12- (D) months of age. The grey bars represent control mice and red bars nMlkl-KI mice that were normalized to β-actin/β-microglobulin and expressed as fold change. Data were obtained from 6-month-old control mice (n = 5), 6-month-old nMlkl-KI mice (n = 4), 12-month-old control mice (n = 6), and 12-month-old nMlkl-KI mice (n = 6). The data represent the mean ± SEM. Group comparisons were performed using the non-parametric Mann-Whitney U test. To control the false discovery rate (FDR) across multiple tests (9-11 genes), p-values were adjusted using the Benjamini-Hochberg procedure. **p ≤ 0.01, *p ≤ 0.05.

To determine how overexpressing MLKL in the brain impacted necroptosis, we measured the levels of the necroptotic proteins (RIPK1, RIPK3 and MLKL) and MLKL-oligomers (marker of necroptosis) in the cortex and hippocampus of 6-month-old male mice. We focused on male mice because almost all the previous research on necroptosis in the brain used male subjects, and therefore, we knew necroptosis occurred normally in male mice. As shown in Figure 2A & B, the levels of MLKL (monomers) were significantly elevated in the cortex (~7-fold, Fig. 2D) and hippocampus (~3-fold, Fig. 2E) of nMlkl-KI mice compared to control mice. The levels of RIPK1 and RIPK3 were not significantly altered in the cortex and hippocampus of the nMlkl-KI mice. Importantly, we found that necroptosis was induced in the nMlkl-KI mice as shown by the presence of MLKL-oligomers in the cortex and hippocampus. MLKL-oligomer levels were 3- to 4-fold (Fig. 2G & H) higher in the cortex and hippocampus of the 6-month-old mice nMlkl-KI mice compared to the control mice. To investigate whether the MLKL transgene continues to be expressed in neurons at later stages of aging, we analyzed MLKL expression in the cortex of 12-month-old mice. We found that MLKL continued to be overexpressed (~5-fold, Fig. 2F) in the cortex of the old nMlkl-KI mice, which was again associated with increased necroptosis as shown by the increase in MLKL-oligomers (Fig. 2I).

### Neuron-specific overexpression of MLKL is associated with neuroinflammation

Necroptosis is an inflammatory cell death pathway; therefore, we studied the impact of MLKL overexpression on neuroinflammation by measuring the transcript levels of key inflammatory markers (Fig. 3). Because a limited amount of hippocampus was available for analysis, these experiments were conducted in only the cortex. At 6-months of age, nMlkl-KI mice exhibited a significant increase in transcripts for inflammatory markers such as Iba-1, F4/80, MHC-II, MCP-1, Tnfr1, Il-6 and Cxcl-8 (Fig. 3A, B). This inflammatory response in nMlkl-KI mice persisted at 12-months when a significant increase in transcripts for GFAP, Tnf-a, Cxcl1, Cxcl8, Cxcl10, and Mmp-12 were observed (Fig. 3C, D).

**Figure 4.**
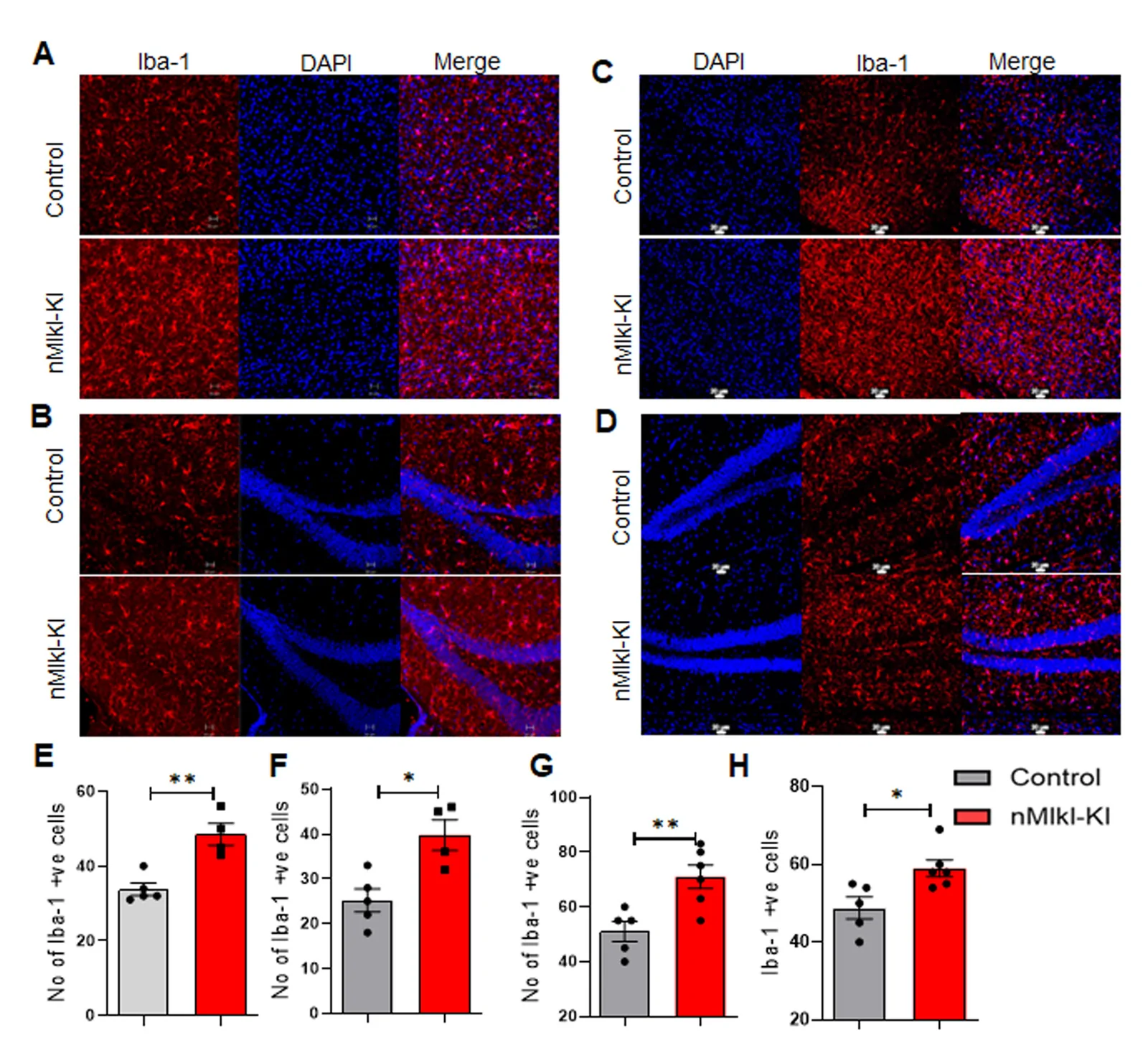
**Neuronal MLKL over expression induces microglial proliferation in the brain**. The expression of Iba-1 was detected by immunofluorescence in the cortex and hippocampus of control and nMlkl-KI mice. Representative confocal images show the expression of Iba-1 (red) and DAPI (blue) in the cortex layer V (A) and hippocampus dentate gyrus region (B) of 6-month-old mice and the cortex layer V (C) and hippocampus dentate gyrus region (D) of 12-month-old mice. Graphical representation of number of Iba-1 positive cells is shown for cortex (E) and hippocampus dentate gyrus region (F) at 6-months of age and cortex (G) and hippocampus dentate gyrus region (H) at 12-months of age. The grey bars represent control mice and red bars represent nMlkl-KI mice. Data were obtained from 5 mice per group and are expressed as the mean ± SEM. Group comparisons were performed using the non-parametric Mann-Whitney U test. **p≤ 0.01, *p≤ 0.05. Scale bar: 20 μm.

To evaluate the effect of MLKL overexpression on microglial activation and proliferation, which are markers of neuroinflammation, we performed immuno-fluorescence analysis of Iba-1 expressing cells in the cortex and hippocampus of nMlkl-KI and control mice. Representative confocal images show the distribution and intensity of Iba-1-positive microglia in the cortex (Fig. 4A) and the dentate gyrus region of the hippocampus (Fig. 4B) of 6-month-old mice, as well as in the cortex (Fig. 4C) and dentate gyrus region of the hippocampus (Fig. 4D) of 12-month-old mice. Quantification of Iba-1-positive microglial cells showed a significant increase in microglial numbers in the cortex (Fig. 4E) and dentate gyrus region of the hippocampus (Fig. 4F) of 6-month-old mice treated with tamoxifen. This was also observed in 12-month-old mice, with a significant increase in the number of Iba-1 positive microglia in the cortex (Fig. 4G) and dentate gyrus region of the hippocampus (Fig. 4H).

We also assessed astrogliosis in the hippocampal regions of the nMlkl-KI mice at 6- and 12-months of age by assessing number of GFAP+ astrocytes using immunofluorescence. No difference was observed in astrogliosis in hippocampus of the nMlkl-KI compared to control mice (Supplementary Fig. 2).

**Figure 5.**
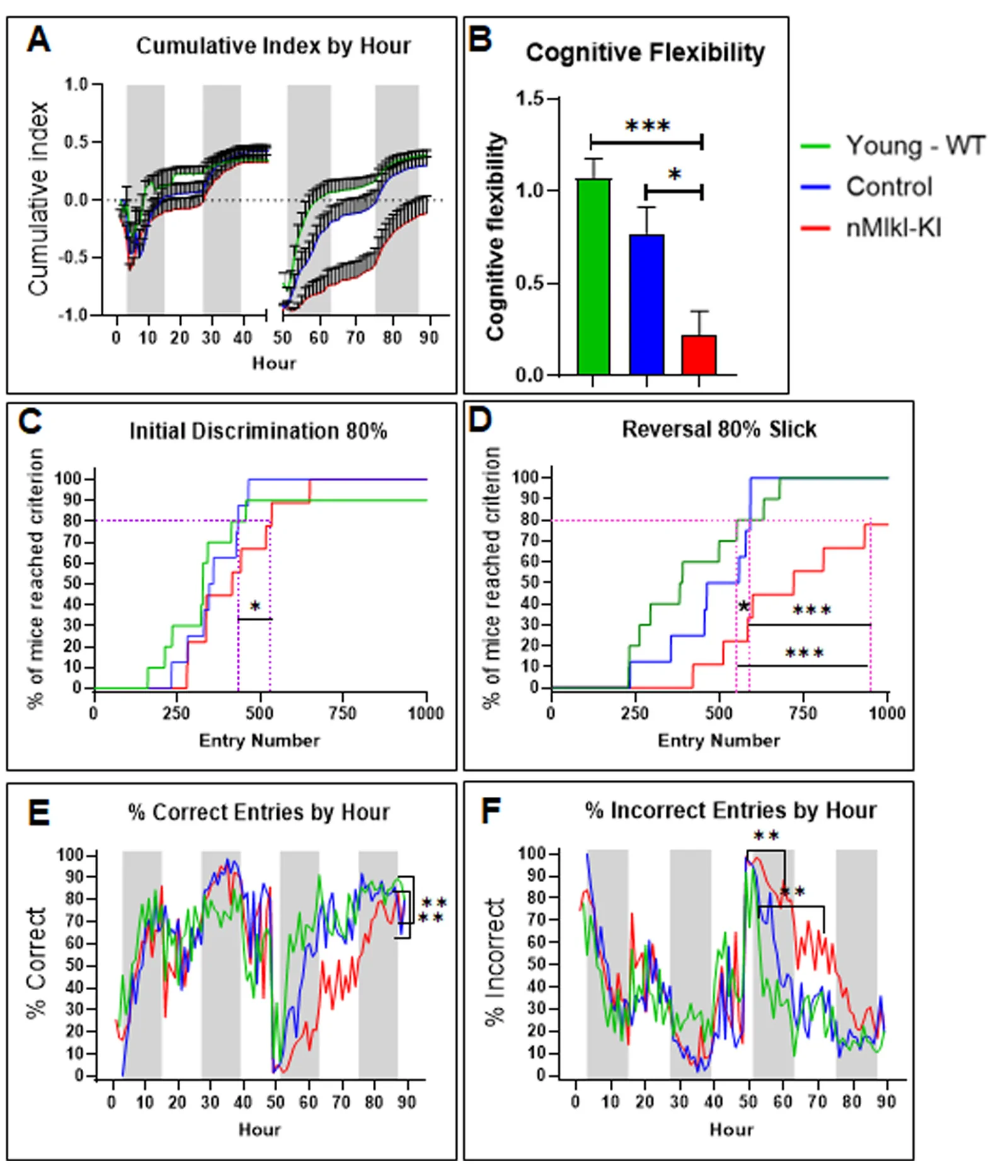
**Neuronal specific MLKL over expression is associated with cognitive decline**. Using the home cage Noldus PhenoTyper, cognition was measured in 6-month-old WT mice (green, n=9) and 12-month-old control (blue, n=6) or nMlkl-KI (red, n=9) mice. Panel A shows the cognitive performance as measured by cumulative learning, which represents the average learning index for each group over consecutive hours with the gray shaded areas showing the dark phase. Panel B shows the decrease in cognitive flexibility (during hours 51-90 of the reversal phase) in nMlkl-KI mice compared to control and young WT mice. Panels C and D show the required steps to reach an 80% success rate during the initial discrimination phase and the reversal learning phase, respectively. Panels E and F show the percentage of correct and incorrect entries, respectively over the entire experiment. Shaded bars in graphs A, E and F represent the dark periods of the Light:Dark cycle. The data are presented as mean ± SEM. * p ≤ 0.05, **p≤ 0.01, ***p≤ 0.001.

### Neuronal-specific MLKL overexpression induced cognitive impairment in nMlkl-KI mice.

Because neuroinflammation is often associated with changes in cognition [42, 43], we measured the cognitive status of 12-month-old nMlkl-KI mice using an Ethovision Phenotyper. The data in Figure 5 show various measures of cognitive performance of 12-month-old nMlkl-KI and control mice and young (6-months) wildtype (WT) mice, which were used as a reference, control. The data in Figure 5A show the cumulative index, which represents the average learning index for each group over 45 hours when cognition was measured. No significant difference was found in the three groups of mice for the initial discrimination learning, showing that overexpressing MLKL did not impact the animals desire for food, which could impact cognition measured by the Phenotyper. We also observed no change in food consumption of the control and nMlkl-KI mice (1.8 ± 0.1mg/day). In addition, we observed no significant differences in locomotor activity, distance moved, or the circadian rhythm in activity in the two groups (Supplementary Fig. 3A). However, the cumulative learning index during the reversal phase was dramatically reduced in the nMlkl-KI mice compared to the other two control groups. The cognitive data were also measured as cognitive flexibility, which is the ability to adapt cognition and behavior in response to changing environment or task demands. As shown in Fig. 5B, cognitive flexibility was significantly reduced (67%) in the nMlkl-KI mice compared to the two control groups. When the steps required to reach 80% success rate were measured for the initial discrimination phase (Fig. 5C) and the reversal learning phase (Fig. 5D), we again observed that the nMlkl-KI mice exhibited a pronounced deficit in their ability to reach the 80% success especially during the reversal phase when the nMlkl-KI mice failed to reach this criterion even after 1,000 entries. The percentage of correct (Fig. 5E and Supplementary Fig. 3B) and incorrect (Fig. 5F) entries during both learning phases was also analyzed. A significantly lower percentage of correct entries, and a higher percentage of incorrect entries were observed in the nMlkl-KI mice in the reversal phase of the analysis compared the two control groups. These findings demonstrate that the overexpression of MLKL in neurons impaired the cognition of nMlkl-KI mice.

**Figure 6.**
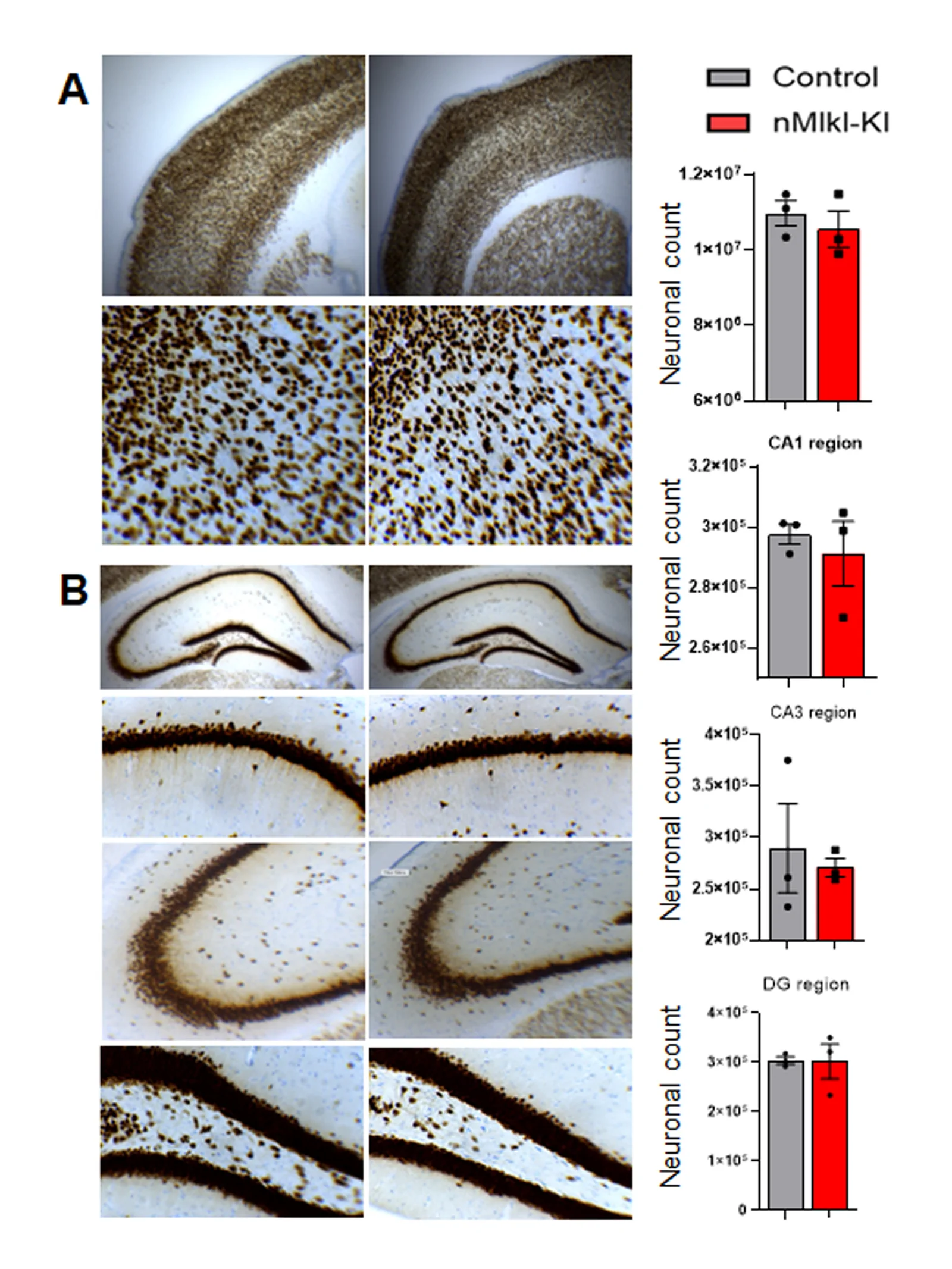
**Neuronal density did not change in the brains of nMlkl-KI mice**. Using unbiased stereology, neuronal density was measured in the cortex and hippocampus of 12-month-old control and nMlkl-KI mice by SRC Biosciences (Tampa, FL). Neurons were stained with NeuN (brown) and counterstained with Nissl. Panel A shows representative images for the neocortex region at 40x (upper row) and 200x (lower row) magnification. Panel B shows representative images from the entire hippocampus (upper row; 40x magnification) and the CA1 (middle row; 200x magnification), CA3 (bottom row; 100x magnification) and dentate gyrus (bottom row; 200x magnification) regions. The graphs on the right represent total neuronal counts for the cortex and the CA1, CA3 and dentate gyrus regions of the hippocampus for control (grey) and nMlkl-KI (red) mice. Data were obtained from 3 mice per group and are expressed as the mean ± SEM. Group comparisons were performed using the non-parametric Mann-Whitney U test.

**Figure 7.**
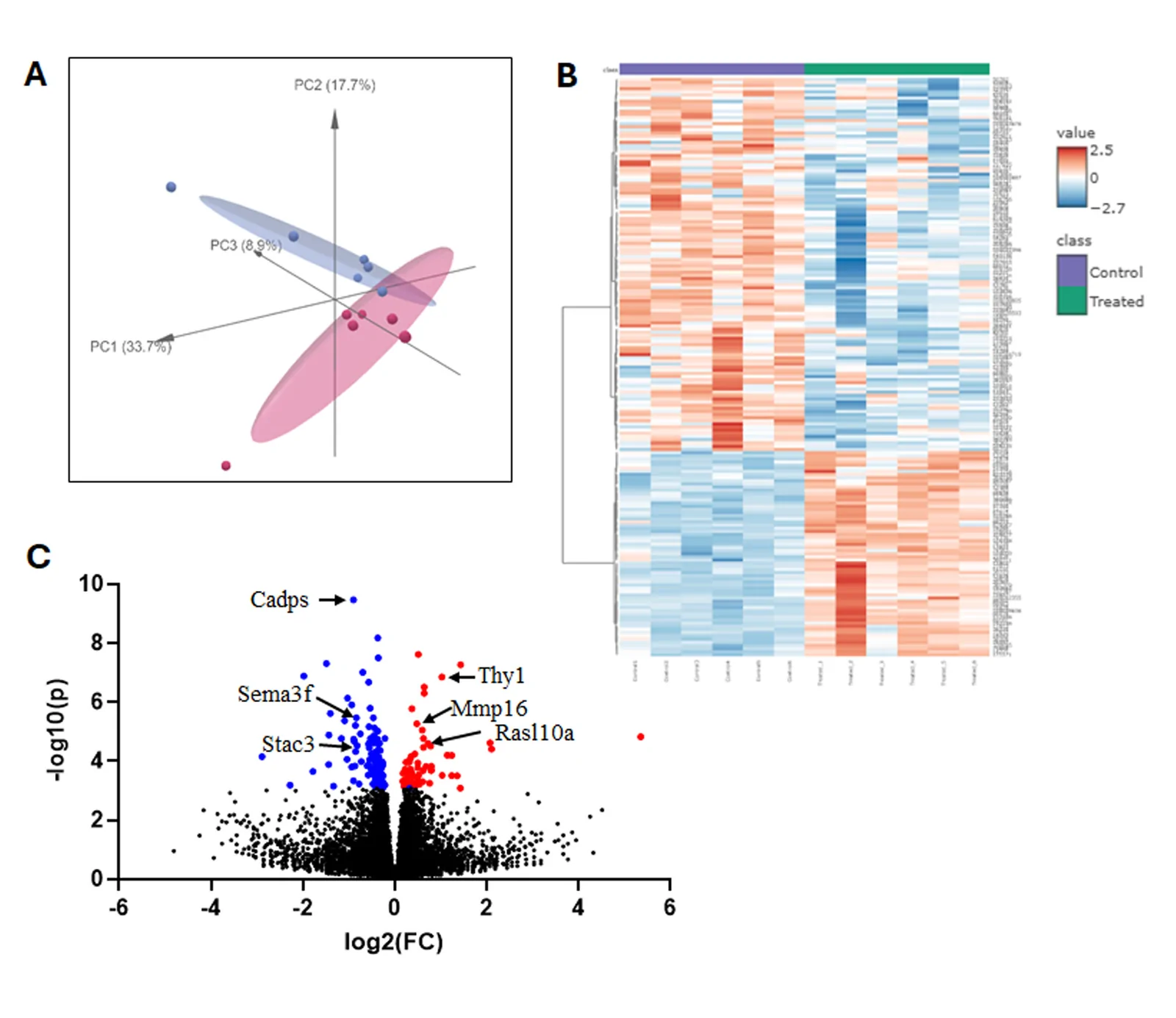
**MLKL over expression in neurons alters the transcriptome of the cortex**. At 12-months of age, differentially expressed genes (DEGs) in the cortex were identified by RNA-seq analysis. Panel A shows the Principal Coordinates Analysis (PCoA) sample clustering based on gene expression profiles. Panel B shows the heatmaps generated using normalized log-transformed counts of the 184 DEGs across in each of the mice studied. Gene expression values were normalized and clustered using hierarchical clustering. Color intensity represents expression levels, with red indicating high expression and blue indicating low expression. Panel C shows the volcano plot of the DEGs for the nMlkl-KI mice compared to the control mice. DEGs (adjusted p < 0.1, |log_2_FC| ≥ 1) are highlighted in red (upregulated) and blue (downregulated), while non-significant genes are shown in black. A complete list of the 184 DEGs is provided in Supplementary Table 1. Data were obtained from 6 mice per group.

### Neuronal-specific MLKL overexpression does not lead to a loss of neurons in the hippocampus and cortex.

Because necroptosis is a regulated form of cell death [44, 45] and because neuronal loss can lead to a decline in cognition [25, 46], we determined if neuronal loss was responsible for the reduced cognition observed in the nMlkl-KI mice using unbiased stereology to measure neuronal density. Stereological quantification was performed in both the cortex and hippocampus of 12-month-old nMlkl-KI and control mice. Figure 6 presents representative images of neuronal density in the cortex and hippocampus. Quantitative analysis revealed no significant difference in neuronal density between control and nMlkl-KI mice in either the cortex or hippocampus. These data indicate that while MLKL overexpression in neurons impaired cognition, this effect is not attributable to a significant loss of neurons in the cortex or hippocampus.

### The impact of MLKL overexpression in neurons on the transcriptome of the cortex.

To gain a better understanding of potential mechanisms by which neuronal MLKL overexpression contributes to cognitive decline without neuronal death in nMlkl-KI mice, we measured the transcriptome of brains from 12-month-old nMlkl-KI and control mice. Because all the hippocampus was used for measuring neuronal density and immunofluorescence analyses, it was not available for RNA-seq analysis. Therefore, our study focused on the transcriptome of the cortex. Principal Coordinates Analysis (PCoA) of the RNAseq data showed a clear clustering of samples based on global gene expression profiles with a separation between control and nMlkl-KI mice (Fig. 7A). These data indicate that the transcriptome of the cortex is likely to be altered by overexpressing MLKL in neurons. We identified a total of 184 differentially expressed genes (DEGs) that changed significantly (adjusted p < 0.1 and |log2FC| ≥ 1) in the nMlkl-KI mice compared to the control mice, and these DEGs are listed in Supplementary Table 1. Figure 7B presents a heat map illustrating the expression patterns of the normalized log counts of differentially expressed genes (DEGs) in the six control and six nMlkl-KI mice studied. The volcano plot (Fig. 7B) shows the 184 DEGs (65 upregulated and 119 down regulated) based on statistical significance and magnitude of expression change. Some of the key DEGs that could be involved in neuronal dysfunction include: Cadps, Sema3f, and Celsr3, which are associated with synaptic function and neurotransmission [47-50]; Stac3 and Cadps, which play roles in calcium signaling and neuronal excitability [47-51]; and Mmp16 and Mill2, which contribute to extracellular matrix remodeling and cell adhesion [52]. In addition, Neurl1a is implicated in protein ubiquitination and synaptic plasticity [53], Celsr3 is involved in neuron axon guidance [50], Dok6 is a key regulator of signaling receptor binding activity [54], whereas Arhgdia is linked to the regulation of the synaptic vesicle cycle and the semaphorin-plexin signaling pathway [55]. Several other genes are associated with behavioral changes, e.g., Cnrip1 [56], Gipr [57] and Tnrc6a [58].

**Figure 8.**
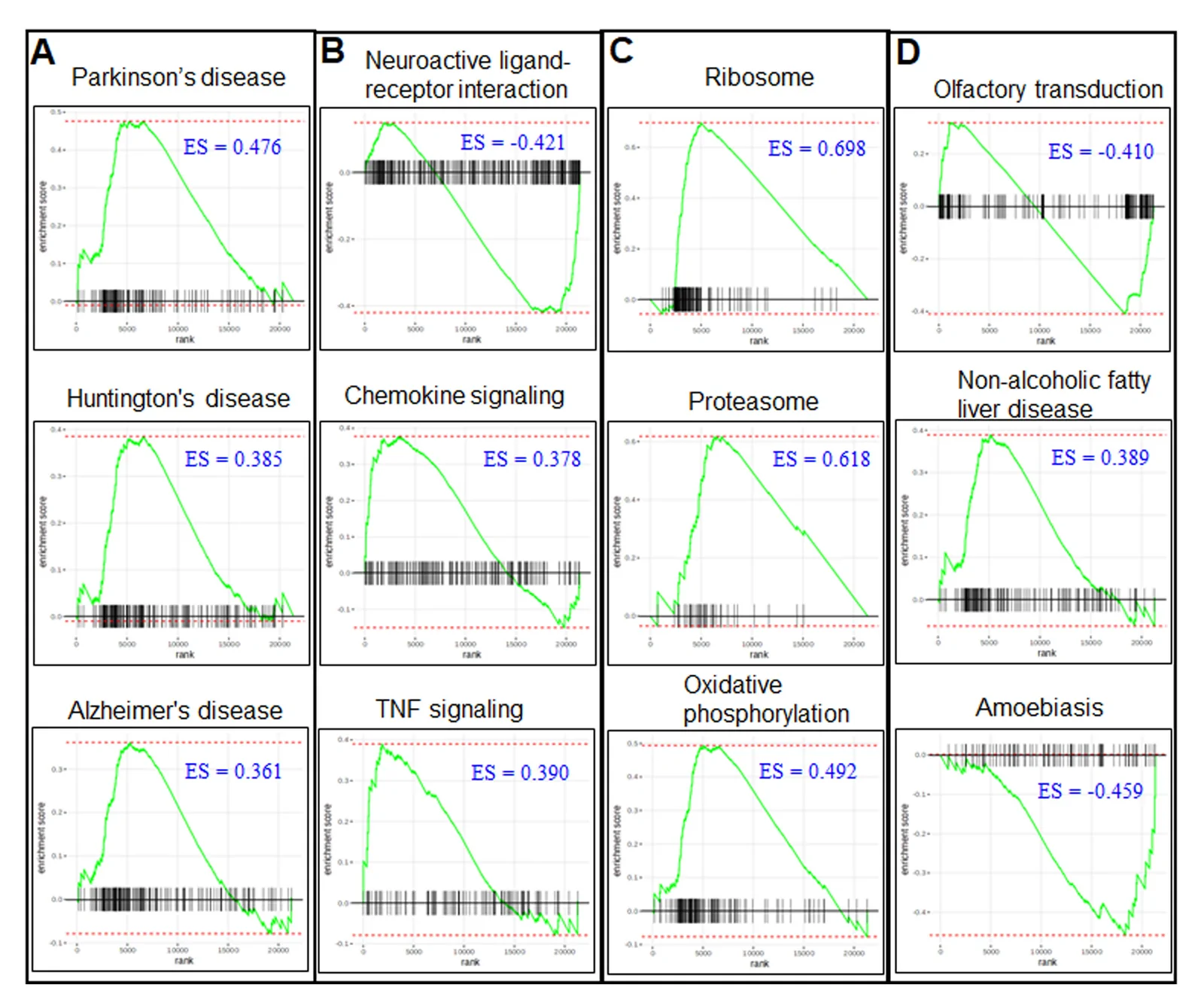
**Neurodegenerative and inflammatory pathways are significantly enriched in the cortex of nMlkl-KI mice**. Gene Set Enrichment Analysis (GSEA) plots are presented showing significantly enriched pathways (listed in Supplementary Table 2) in 12- month-old nMlkl-KI mice compared to the control mice. The green curve represents the enrichment score (ES), depicting the cumulative distribution of ranked genes and highlighting the degree to which the gene set is overrepresented at the top (upregulated) or bottom (downregulated) of the ranked list. Vertical bars along the x-axis indicate the positions of genes from the gene set within the ranked list. A positive ES indicates an activation of the pathway in the nMlkl-KI mice while a negative ES indicates a down regulation of the pathway in the nMlkl-KI mice compared to control mice. The panels display the enrichment for neurodegenerative pathways (A), signaling pathways (B), proteostasis and bioenergetics pathways (C) and organismal systems and disease pathways (D). Data were obtained from 6 mice per group.

To identify potential biological pathways that could be altered in the cortex by overexpressing MLKL in neurons, we performed Gene Set Enrichment Analysis (GSEA) using KEGG (Kyoto Encyclopedia of Genes and Genomes) pathways. Supplementary Table 2 lists the 12 KEGG pathways that showed significant changes (p < 0.05) in enrichment in the nMlkl-KI mice compared to control mice, and Figure 8 shows the significant enrichment in these biological pathways in the nMlkl-KI mice. Notably, neurodegenerative pathways, including Parkinson’s disease, Huntington’s disease, and Alzheimer’s disease were all enriched in the nMlkl-KI mice, suggesting the overexpression of MLKL in neurons leads to a similar neuronal dysfunction found in these neurodegenerative diseases (Fig. 8A). In addition, several signaling pathways exhibited significant alterations, e.g., the neuroactive ligand-receptor interaction pathway was downregulated, while the chemokine signaling and TNF signaling pathways were upregulated (Fig. 8B). In addition, pathways related to proteostasis and bioenergetics (Fig. 8C), including ribosome and proteasome function (protein homeostasis) and oxidative phosphorylation (cellular energy metabolism), were upregulated, suggesting that disruptions in these essential cellular processes could contribute to neuronal dysfunction. Finally, organismal systems and disease-related pathways (Fig. 8D) were also enriched, indicating broader physiological and pathological implications for overexpressing MLKL in the neurons of nMlkl-KI mice. For example, olfactory dysfunction has been linked to neurodegenerative diseases [59, 60] and non-alcoholic fatty liver disease shares common risk factors with neurodegeneration, e.g., inflammation, oxidative stress, and metabolic syndrome [61, 62]. Because we used bulk RNA from the cortex for our transcriptomic data, it lacks cellular resolution and making it difficult to determine the cell type-specific transcriptional changes that are occurring in the cortex of the nMlkl-KI mice.

## DISCUSSION

Several lines of research over the past eight years demonstrate that neurons are particularly vulnerable to necroptosis. In 2017, Caccamo et al. [12] reported that markers of necroptosis (e.g., phosphorylated-MLKL, P-MLKL) were increased in the brains of patients with Alzheimer’s disease (AD) and that most of the P-MLKL immunoreactivity colocalized with neurons. They also found that markers of necroptosis were colocalized to neurons in the brains of AD-transgenic mice. Subsequently, Koper et al. [14] and Jayaraman et al. [63] also found that the immunoreactivity to markers of necroptosis colocalized to neurons in patients with AD. Markers of necroptosis have also been reported to colocalize to neurons in patients with Parkinson’s disease [15], multiple sclerosis [64], and ischemic brain injury [65]. We also observed that the age-related increase in necroptosis in the brains of mice occurred primarily in neurons with ~80% of the immunoreactivity of P-MLKL colocalizing to neurons and less than 10% with microglial cells and no immunoreactivity detected in astrocytes [7]. Our study with Neuro-2A cells, in which MLKL was overexpressed without cell death, showed that MLKL strongly attenuated Ca^2+^ dynamics in response to inomycin in the presence of milimolar Ca^2+^ levels in the bathing solution, or after Ca^2+^ removal and restoration [26]. These data suggest that increased expression of MLKL in neurons can result in either higher Ca^2+^ buffering, extrusion, or intracellular compart-mentalization. Because altered neuronal calcium homeostasis is associated with cognitive deficits in normal aging [66] and AD [67, 68], we hypothesized that the neuron-specific necroptosis could play a role in the etiology of cognitive aging. The purpose of this study was to determine the *in vivo* impact of overexpressing MLKL in neurons of young/adult mice on brain function when necroptosis and neuroinflammation were minimal.

To determine the impact of overexpressing MLKL in neurons in the absence of aging or neurodegeneration, we used Mlkl-KI mice crossed to Slick-H Cre mice, in which MLKL expression was induced 3- to 7-fold in the hippocampus and cortex, respectively, at 2.5-months of age. At 6-months, the increase in MLKL levels was associated with an increase in MLKL-oligomerization (marker of necroptosis) and markers of inflammation in the cortex and hippocampus, including microglial activation and proliferation, a marker of neuroinflammation. To measure the impact of overexpressing MLKL in neurons on brain function, we measured cognition using the Noldus PhenoTyper, which allows one to measure cognition during both the dark and light phases in a home-cage setting. This gives one a more accurate measure of cognition because cognition is measured during the dark phase when the mice are normally active [69]. Logan et al. [28] showed that both initial discrimination and reversal learning decreased in old mice. For example, male C57BL/6J mice over 20-months of age required more entries to reach an 80% success rate in the initial phase of cognitive analysis and were severely impaired in the reversal phase. In our study, the young adult (6-months) WT and the older (12-months) control and nMlkl-KI mice performed similarly during the initial phase with all groups reaching the 80% success criterion at comparable rates, which was expected based on previous research showing that the age-related decline in cognition was observed in mice only when they were over 20-months of age [28]. However, in the reversal phase, approximately 50% of the nMlkl-KI mice failed to meet the 80% success criterion in contrast to 6-month-old WT and the 12-month-old control mice, which showed better performance in adapting to the reversal task. Cognitive flexibility was also reduced in the 12-month-old nMlkl-KI mice compared to the 12-month-old control mice, resulting in a longer time to reach the criterion.

An interesting observation from our study was that the increased neuroinflammation and loss of cognition in the nMlkl-KI mice occurred in the absence of any significant loss of neuronal cells in the cortex and hippocampus even though necroptosis had been induced in neurons in these brain regions. While it is generally assumed that necroptosis triggers cell death by the rupture of plasma membrane, recent studies show that cells can avoid necroptosis induced cell death through the ESCRT-III (endosomal sorting complex required for transport III) system, which prevents cell rupture and death [70, 71]. MLKL (phosphorylated and oligomers) has been shown to interact with ESCRT proteins and flotillins and to be released from cells through extracellular vesicles (EVs) along with MLKL-damaged membranes thereby maintaining membrane integrity and preventing cell death. Jayaraman et al. [63] showed that the expression of necroptosis markers and ESCRT III proteins are elevated in both the post-mortem AD brain and human iPSC neurons in response to TNF, and we found that Neuro-2A cells overexpressing MLKL released EVs containing MLKL [26]. In nMlkl-KI mice, we detected increased transcript levels of extracellular vesicles markers VPS-4B and CHAMP-2B (Supplementary Fig. 4), which is consistent with the possibility that packaging MLKL into EVs prevented neuronal loss in the nMlkl-KI mice. The increase in neuroinflammation we observe could be due to the necroptotic EVs released by the neurons. While it is generally assumed that necroptosis triggers inflammation when DAMPs released by the rupture of the plasma membrane activate the innate immune system (e.g., macrophages and microglia) [72, 73], Shlomovitz et al. [74] showed that necroptotic EVs phagocytosed by macrophages triggers the secretion of pro-inflammatory cytokines and chemokines. Therefore, it is possible that the increased neuroinflammation we observed in the cortex and hippocampus of the nMlkl-KI mice in the absence of neuronal cell death was due to EVs released from neurons being phagocytosed by microglia.

To gain insight into the mechanism of how overexpressing MLKL in neurons in the absence of neuronal death could affect brain function of the nMlkl-KI mice, we measured the transcriptome of the cortex. Unbiased pathway analysis of the transcriptome data showed the upregulation of pathways involved in neurodegeneration in the nMlkl-KI mice, e.g., Alzheimer’s, Parkinson’s, and Huntington's disease pathways. These data suggest that increased necroptosis in neurons, which has been reported in Alzheimer’s [14, 63], Parkinson’s [15] and Huntington [19] disease, might contribute to neurodegeneration through a mechanism(s) other than neuronal loss. One mechanism could be the down regulation of the neuroactive ligand-receptor interaction pathway, which was observed in the nMlkl-KI mice. This pathway encompasses a broad spectrum of neurotransmitter receptors (e.g., glutamate, GABA, dopamine, serotonin, acetylcholine) and their corresponding ligands, which mediate key signaling events in the central nervous system [75]. Downregulation of this pathway has been shown to lead to a disruption of neurotransmission that is associated with a decline in cognitive [76, 77]. In addition, the upregulation of the ribosome pathway observed in the nMlkl-KI mice could also play a role in the decline in cognition in the nMlkl-KI mice. The dysregulation of ribosomal biogenesis has been associated with neurodegenerative diseases such as Alzheimer's, Huntington's, and Parkinson's diseases [78, 79]. Aberrant ribosomal biogenesis can also activate cellular stress responses, particularly the ribosomal stress pathway mediated by TP53, which could impair neuronal function [80, 81]. The pathway analysis of the transcriptome also supports our data showing increased neuroinflammation in the brains of the nMlkl-KI mice. For example, the cytokine signaling and TNF signaling pathways were increased in the nMlkl-KI cortex. Chronic neuroinflammation, driven by sustained activation of these two signaling pathway, is increasingly recognized as a key mechanism underlying synaptic dysfunction, neuronal damage, and impaired neuroplasticity [82].

A limitation to our study was that we overexpressed MILK in neurons using Slick-H Cre (Thy1-CreERT2) transgenic mice, which is a pan-neuronal driver that induces MLKL expression in multiple brain regions, including the substantia nigra and locus coeruleus. These areas, which are also involved in learning, memory, and age-related cognitive decline, were not specifically examined in this study. Based on our research, future studies should use region-specific genetic targeting to determine the impact of overexpressing MLKL in specific brain areas on neuroinflammation and cognition. In addition, because we focused on male mice, future experiments should determine if overexpressing MLKL in neurons of female mice has a similar impact on neuroinflammation and cognition because changes in neuroinflammation and cognition have been shown to differ in males and females [83-85].

Based on our data, we propose the model presented in Figure 9 to explain how overexpressing MLKL in neurons could impact brain function and cognition in the absence of neuronal death. The increase in MLKL in neurons results in the initiation of necroptosis and formation of MLKL-oligomers, which are extruded from the neurons in EVs by the ESCART-III pathway, thereby preventing neuronal death. The EVs released by the neurons are phagocytosed by microglia leading to neuroinflammation [42, 43, 86]. For example, activated microglia produce high levels of pro-inflammatory cytokines (e.g., TNF-α, IL-1β, IL-6), chemokines (e.g.,Ccl2, Ccl5, Cxcl1) and inflammatory mediators (e.g. reactive oxygen species, nitic oxide) that exacerbate inflammation and contribute to synaptic dysfunction and impair cognition [86, 87].

Alternatively, cognitive decline in the nMlkl-KI mice could also arise from neuronal dysfunction as a result of the increased levels of MLKL in the neurons. We propose that the presence of high levels of MLKL monomers and oligomers in the neuron before the oligomers are extruded from the neuron generates a proteotoxic stress leading to alterations in gene expression that impairs neuronal function. Previous studies have shown prolonged overexpression of proteins can lead to the accumulation of misfolded proteins and aggregation of damage proteins causing cellular stress [88-90]. Our transcriptomic data are consistent with the increased MLKL monomers/oligomers leading to changes in gene expression in the cortex. For example, the three neurodegenerative pathways that involve protein misfolding and aggregation are increased in the nMlkl-KI mice. In addition, the proteasome pathway is up-regulated in the cortex of the nMlkl-KI mice that is likely in response to the increased demand for the proteasome degradation of MLKL to minimize misfolding and aggregation [91].

Our transcriptome data are consistent with our previous observation that overexpressing MLKL in neurons in culture resulted in Ca^2+^ dyshomeostasis [26]. For example, we found that inducing MLKL in neurons *in vivo* significantly reduced the expression of two genes (Stac3 and Cadps) that are involved in calcium fluxes into the cytosol [47, 51, 92]. Stac3 has been shown bind to L-type voltage-gated Ca^2+^ channels and delay their inactivation, thus allowing more Ca^2+^ influx into the cells [92]. Stac3 is also crucial for the coupling between L-type Ca^2+^ channels and Ryanodine receptors in the ER, which mediate Ca^2+^ release from this intracellular store [93]. Cadps is a key regulator of calcium-mediated signaling, particularly in the context of vesicle exocytosis. It contributes to the assembly of the SNARE complex, a critical component in the fusion of secretory vesicles with the plasma membrane, thereby facilitating neurotransmitter release [47]. Data from our previous study suggested that overexpressing MLKL in neuronal cells increases the retention of Ca²^+^ in the endoplasmic reticulum (ER), which is revealed by larger elevations in the cytosolic Ca^2+^ level when forcing the Ca^2+^ emptying from the ER with the drug thapsigargin [26]. However, our Stac3 data suggests that the machinery necessary to mobilize these Ca^2+^ reservoirs under physiological conditions may not be fully available in MLKL expressing neurons. Moreover, our previous study also showed reduced cytosolic and mitochondrial Ca²^+^ levels in response to Ca^2+^ fluxes through the plasma membrane [26]. Importantly, reduced mitochondrial Ca^2+^ uptake in neurons can impair the activation of the tricarboxylic acid (TCA) cycle and oxidative phosphorylation (OXPHOS) [94]. Because changes in Ca^2+^ flux in the mitochondria can impair ATP synthesis [95, 96], we propose in Figure 9, that changes in Ca²^+^ influx and homeostasis induced by changes in gene expression in response to MLKL overexpression could impair cognition through changes in neuronal bioenergetics.

Increased MLKL expression in neurons could also directly impair neuronal function and cognition through changes in the expression of genes involved in neuronal signaling. For example, we found that the neuroactive ligand-receptor interaction pathway was downregulated in the cortex. In addition, we observed an upregulation of several genes shown to impair synaptic plasticity and lead to neurodegeneration and cognitive deficits, e.g., Neurl1a, Arhgdia, Gabra2, Rasl10a. Excessive Neurl1a activity has been shown to dysregulate Notch signaling and protein turnover [53, 97]. Overexpression of Arhgdia or gabra2 disrupts cytoskeletal remodeling and the balance of inhibitory neurotransmission, respectively, and has been shown to lead to impaired learning and synaptic connectivity [55, 98]. In addition, Rasl10a upregulation alters GTPase signaling, which is vital for NMDA receptor function and has been shown to contribute to brain developmental abnormalities and impaired cognition [99]. Several other genes are altered in the cortex of the nMlkl-KI mice that are associated with behavioral changes. For example, Cnrip1 is increased, and its overexpression in the hippocampus has been associated with psychosis [56]. We also observed a notable downregulation of Gipr and Tnrc6a, both of which have previously been implicated in neurobehavioral and stress-related processes. Down regulation of Gipr can cause behavioral impairments in hippocampus-related tasks, as well as reduced synaptic plasticity in CA1 neurons, and neurogenesis in the hippocampus [57]. Decreased expression of Tnrc6a can lead to anxiety like behaviors reported in the prefrontal cortex of mice exposed to acute restraint stress [58]. Future studies will elucidate how cell-autonomous versus non-cell-autonomous mechanisms of neuronal MLKL accumulation could contribute to cognitive decline in aging. The current study only used 12-month-old mice for transcriptomic and behavioral analyses, based on the previous studies indicating cognitive decline typically occurs after 20 months. Future experiments are planned to specifically measure the neuronal transcriptome from various brain regions of nMlkl-KI mice at multiple ages before 12 months and to correlate these molecular changes with cognitive outcomes.

**Figure 9.**
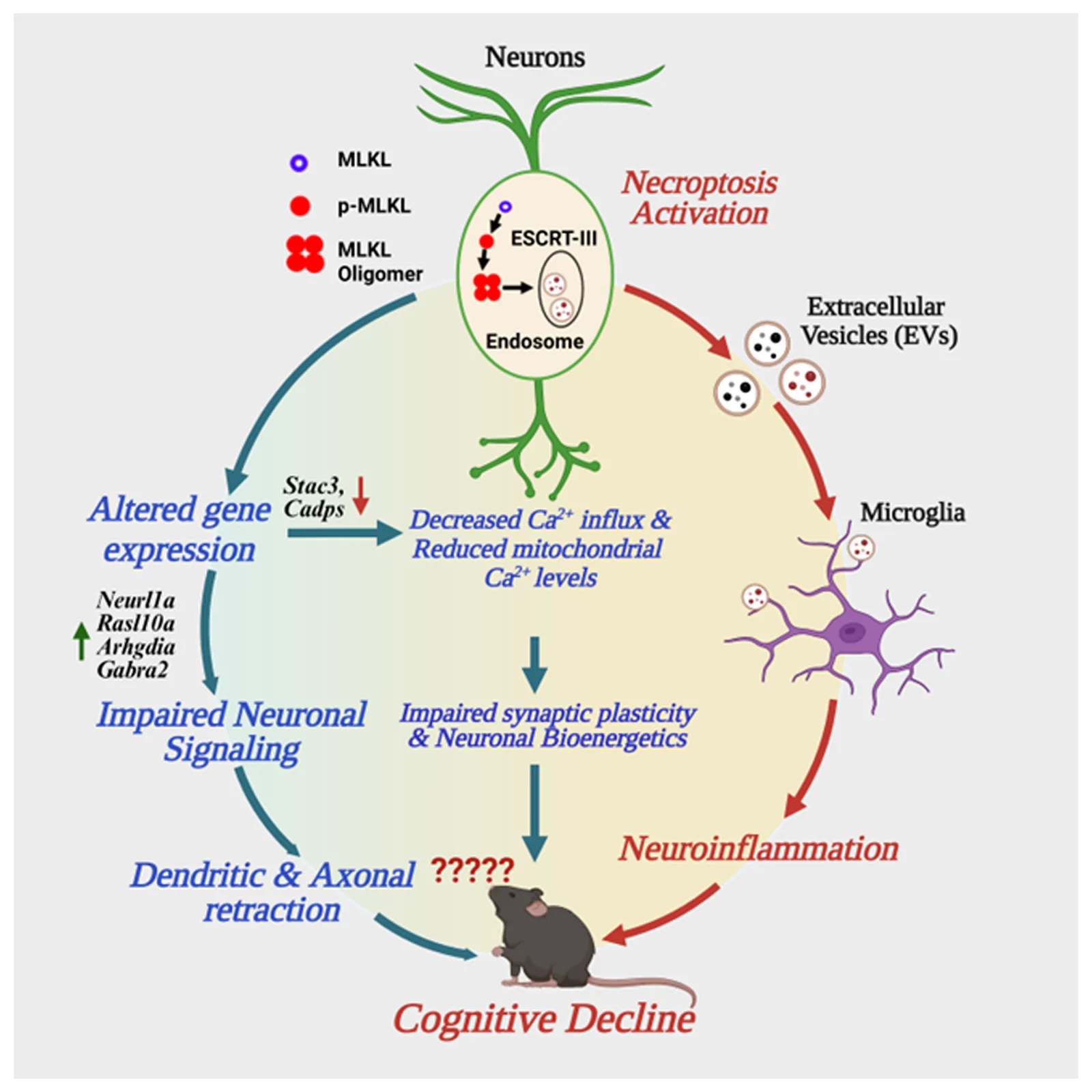
**Proposed model of how MLKL overexpression in neurons affects brain function and cognition in the absence of neuronal cell death**. MLKL overexpression initiates necroptosis and promotes the formation of MLKL oligomers, which are released from neurons via extracellular vesicles (EVs) through the ESCRT-III pathway, preventing neuronal death. Cognitive decline in the nMlkl-KI mice is proposed to arise from three pathways. First as shown by the red arrows, neuroinflammation is triggered by microglia phagocytizing the EVs containing MLKL-oligomers that leads to the release proinflammatory cytokines, chemokines and inflammatory mediators. Second, as shown by the blue arrows, changes in gene expression in neurons induced by elevated levels of MLKL that lead to impaired cognition through two pathways. (1) A decrease in mitochondrial function through the disruption of calcium signaling potentially mediated by the downregulation of Stac3 and Cadps, and (2) Impaired synaptic function that would occur through the up regulation of genes such as Neurl1a, Arhgdia, Gabra2, and Rasl10a. The Figure was created using Biorender software.

## Supplementary Materials

The Supplementary data can be found online at: www.aginganddisease.org/EN/10.14336/AD.2025.0805.

## Data Availability

The RNA sequencing data used in this study is publicly available in the GEO repository under accession number GSE304069.
