# Supplementary material for: Neuronal Necroptosis Drives Neuroinflammation and Cognitive Decline Independent of Neuronal Cell Death
Source: Aging Dis. 2026 Sep 16;17(6):3360–79. doi: 10.14336/AD.2025.0805 (PMC13653739; doi:10.14336/AD.2025.0805)
Supplement: Supplementary file 1 [file AD-17-6-3360-s.pdf]

SUPPLEMENTARY DATA

Neuronal Necroptosis Drives Neuroinflammation and Cognitive Decline Independent of Neuronal Cell Death

Nidheesh Thadathil, Nicholas A Wolf, Roman Wolf, Carlos Manlio Díaz-García, Sreemathi Logan, Daniel B Owen, Kevin Pham, Willard M Freeman, Arlan Richardson

© 2025. Thadathil N et al. Published online at http://www.aginganddisease.org/EN/10.14336/AD.2025.0805

SUPPLEMENTARY DATA

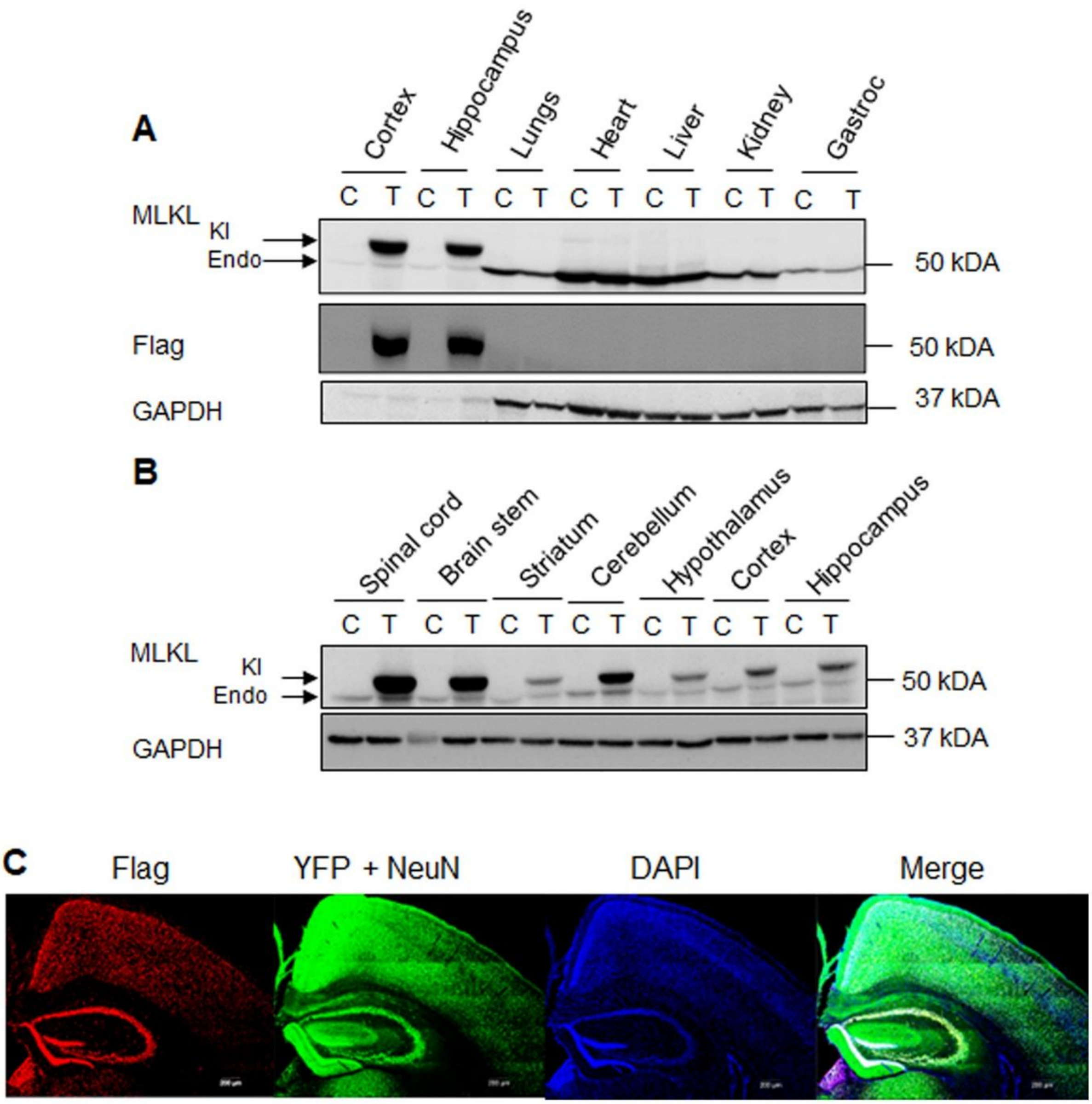

Supplementary Figure 1. **Characterization of MLKL overexpression in nMlkl-KI mice.** Slick H-Cre–Rosa Mlkl mice were treated with corn oil (C, control) or tamoxifen (T) at 45 days of age, and MLKL levels measured at 2.5-months of age. **Panel A** shows western blots of MLKL expression in various tissues from mice using antibodies against Flag (which shows the expression of the Mlkl-transgene) or antibodies against MLKL [which shows the levels endogenous (bottom band) and transgene (top band) expression]. **Panel B** shows the western blots for various brain regions. **Panel C** shows the confocal micrographs of the expression of Flag-tagged MLKL in the entire coronal section.

© 2025. Thadathil N et al. Published online at http://www.aginganddisease.org/EN/10.14336/AD.2025.0805

SUPPLEMENTARY DATA

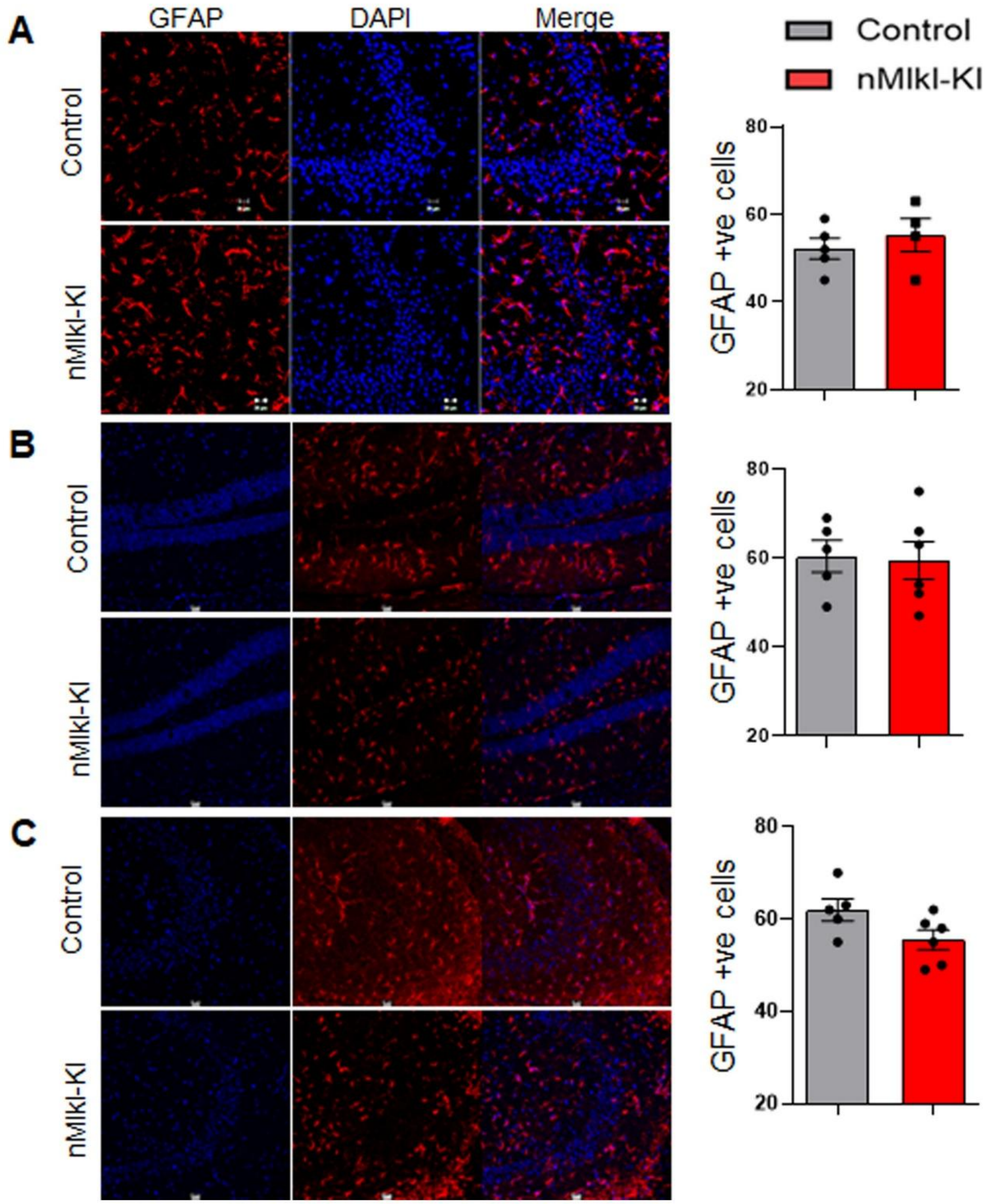

Supplementary Figure 2. **MLKL over expression has no effect on astrogliosis in the hippocampus of nMlkl-KI mice.** The activation of astrocytes (GFAP) in the hippocampus was analyzed by immunofluorescence in 6- and 12- month-old nMlkl-KI and control mice. Representative confocal micrographs show the expression of GFAP (red) and DAPI (blue) in the 6-month-old dentate gyrus **(Panel A)** and CA3 region **(Panel B)**, and 12- month-old CA3 region **(Panel C)**. The graphs on the right show the GFAP positive cells with grey bars representing control mice and red bars representing nMlkl-KI mice. Data were obtained from 5 mice per group and are expressed as the mean ± SEM. Group comparisons were performed using the non-parametric Mann–Whitney U test. Scale bar: 20 µm.

© 2025. Thadathil N et al. Published online at http://www.aginganddisease.org/EN/10.14336/AD.2025.0805

SUPPLEMENTARY DATA

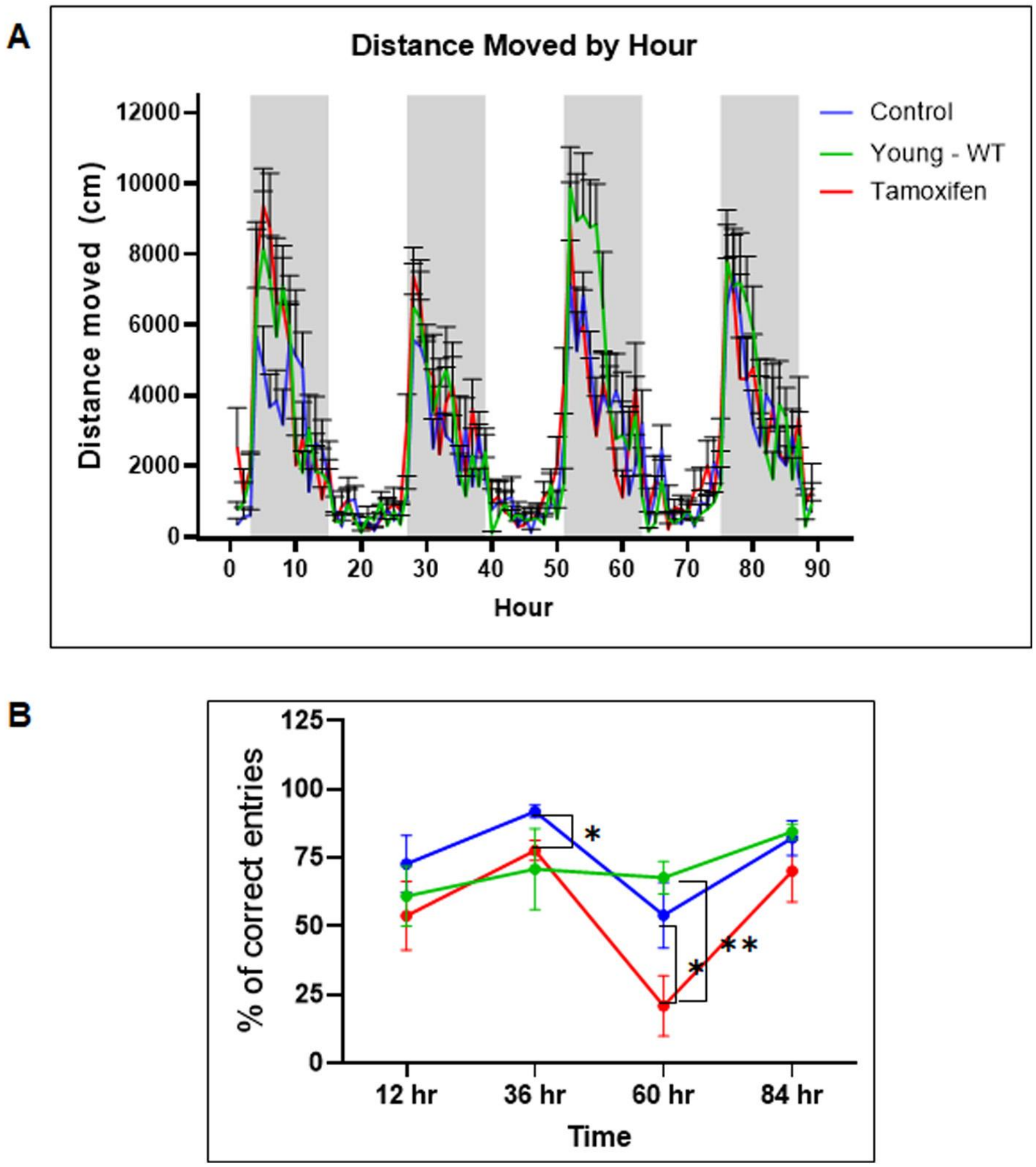

Supplementary Figure 3. **The effect of neuronal specific MLKL over expression on the spontaneous activity and cognition of nMlkl-KI mice**. Using the home cage Phenotyper, various parameters were measured in 6-month-old WT mice (green, n=9) and 12-month-old control (blue, n=6) or nMlkl-KI (red, n=9) mice. **Panel A** shows the spontaneous activity of the mice over the 90 hours. The gray area represents the dark periods of the Light:Dark cycle. **Panel B** shows time course plot of the percentage of correct entries at multiple time points (12, 36, 60, and 84 hours). The data represent the percentage of correct entries at specific time points and are derived from the same dataset used in Figure 5E. The data are presented as mean ± SEM. Statistical analysis for Panel A was performed using one-way ANOVA, and Panel B was analyzed using two-way ANOVA followed by post hoc multiple comparison tests to evaluate group differences. No group differences were observed at 12 hours, while significant differences evident at 60 hours during the reversal phase. *$p \leq 0.05$, **$p \leq 0.01$.

© 2025. Thadathil N et al. Published online at http://www.aginganddisease.org/EN/10.14336/AD.2025.0805

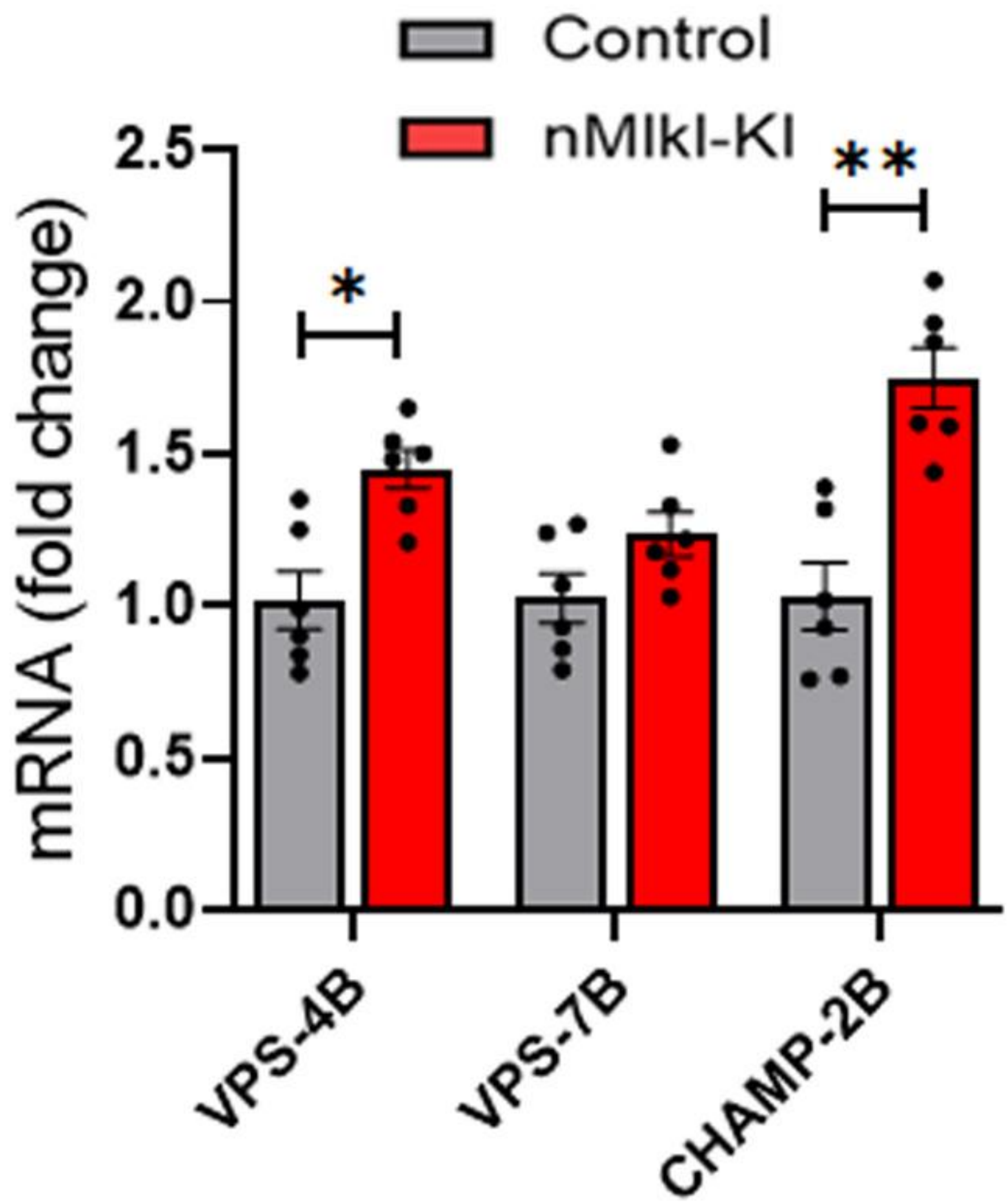

Supplementary Figure 4. The transcript levels of extracellular vesicles markers in the cortex of nMlkl-KI mice. The levels of transcript for extracellular vesicles were measured in the cortex of 6-month-old control (gray bars) and nMlkl-KI (red bars) mice. Data were obtained from 6 mice per group and are expressed as the mean ± SEM. Group comparisons were performed using the non-parametric Mann–Whitney U test. **p≤ 0.01, *p≤ 0.05.

© 2025. Thadathil N et al. Published online at http://www.aginganddisease.org/EN/10.14336/AD.2025.0805

SUPPLEMENTARY DATA

Supplementary Table 1. List of the differentially expressed genes (DEGs) in the cortex of nMlkl-KI mice. The 184 DEGs in the cortex of 12-month-old nMlkl-KI mice that were significantly different compared to control mice are shown. The genes are listed on the basis of their log fold change with the genes most up regulated in the nMlkl-KI mice at the top and those most down regulated at the bottom.

The RNAseq data were analyzed using express analyst software, and adjusted p-values were computed using the Benjamini-Hochberg method to control for false discovery. The Table lists the following for each of the 184 genes: Entrez Gene ID (EntrezID), log fold change (logFC), adjusted p-value (adj.P.Val) and gene symbols (Symbols).

EntrezID	logFC	stat	P.Value	adj.P.Val	Symbols
629967	5.3488	4.3176	1.58E-05	0.009911	Gm11677
613254	2.1063	4.107	4.01E-05	0.01556	AA465934
1E+08	2.0714	4.216	2.49E-05	0.011279	Gm10705
50720	1.4303	5.427	5.73E-08	0.000175	Sacs
13345	1.4258	3.3371	0.000847	0.098789	Twist2
381319	1.3537	3.5936	0.000326	0.054846	Batf3
435766	1.2396	3.9908	6.59E-05	0.021975	Tnni3k
74720	1.2384	3.6011	0.000317	0.053697	Tmem114
20716	1.1388	3.9976	6.40E-05	0.021859	Serpina3n
76293	1.0263	3.6051	0.000312	0.053299	Mfap4
21838	1.0232	5.2624	1.42E-07	0.000304	Thy1
64099	0.79356	3.7095	0.000208	0.044944	Parvg
380711	0.79205	3.7883	0.000152	0.037101	Rap1gap2
74978	0.78584	3.6997	0.000216	0.045724	Lrriq1
75668	0.77532	4.1629	3.14E-05	0.013157	Rasl10a
14395	0.75759	3.4465	0.000568	0.080226	Gabra2
171171	0.71843	4.2116	2.54E-05	0.011279	Ntng2
76220	0.67567	3.7863	0.000153	0.037101	6530402F18Rik
108841	0.63658	5.019	5.19E-07	0.000853	Rdh13
68463	0.63633	5.1153	3.13E-07	0.000557	Mrpl14
14131	0.62409	4.1411	3.46E-05	0.014123	Fcgr3
497097	0.62112	4.2925	1.77E-05	0.009911	Xkr4
269198	0.61333	3.6992	0.000216	0.045724	Nbeal1
66214	0.60858	3.4758	0.000509	0.075517	Rgcc
20723	0.59512	4.4388	9.05E-06	0.007154	Serpinb9
27973	0.52879	3.4255	0.000614	0.081834	Vkorc1
74563	0.51554	3.6144	0.000301	0.05258	Rasgef1c
19290	0.51086	5.5672	2.59E-08	0.000132	Pura
58200	0.49981	3.8525	0.000117	0.032012	Ppp1r1a
623279	0.49787	3.7546	0.000174	0.040735	Dok6
20364	0.49348	3.5419	0.000397	0.064756	Selenow
11853	0.48979	3.481	0.000499	0.075112	Rhoc
17389	0.47767	4.5352	5.75E-06	0.005586	Mmp16
72657	0.4738	3.4148	0.000638	0.08311	Selenoh
237387	0.46904	3.4088	0.000652	0.084324	Lrrc3
57740	0.43697	4.0277	5.63E-05	0.019715	Stk32c
104010	0.4267	3.3859	0.000709	0.089104	Cdh22
18011	0.39287	3.4494	0.000562	0.080226	Neurl1a
14221	0.38789	3.5854	0.000337	0.056153	Fjx1
67815	0.36892	4.7861	1.70E-06	0.002136	Sec14l2
192156	0.36355	3.6464	0.000266	0.051165	Mvd
66078	0.36058	3.6618	0.00025	0.049601	Tsen34
380686	0.35677	3.9728	7.10E-05	0.023337	Cnrip1
72832	0.35468	3.4074	0.000656	0.084324	Crtac1
29858	0.3498	3.5748	0.000351	0.058025	Pmm1

© 2025. Thadathil N et al. Published online at http://www.aginganddisease.org/EN/10.14336/AD.2025.0805

330695	0.33976	3.7262	0.000194	0.043449	Ctxn1
19242	0.33892	3.7271	0.000194	0.043449	Ptn
69161	0.33547	3.6355	0.000277	0.051972	Manbal
103724	0.33122	3.9244	8.69E-05	0.026976	Tbc1d10a
76497	0.3312	3.3761	0.000735	0.090753	Ppp1r11
1E+08	0.31931	3.3375	0.000845	0.098789	Atp6v0c-ps2
16828	0.31363	3.3574	0.000787	0.093693	Ldha
105348	0.31063	3.8618	0.000113	0.031628	Golm1
112406	0.26517	3.5121	0.000445	0.069817	Egln2
18483	0.26463	3.6541	0.000258	0.050102	Palm
192662	0.24671	3.4589	0.000542	0.078792	Arhgdia
13822	0.24045	3.854	0.000116	0.032012	Epb41l2
11772	0.23939	3.5138	0.000442	0.069817	Ap2a2
69202	0.23656	3.4878	0.000487	0.074604	Ptms
11676	0.22754	3.4153	0.000637	0.08311	Aldoc
11803	0.22742	3.5277	0.000419	0.067309	Aplp1
74330	0.22227	3.7205	0.000199	0.043776	Dnajc14
11750	0.19764	3.3826	0.000718	0.089145	Anxa7
68839	0.17762	3.6543	0.000258	0.050102	Ankrd46
319477	0.1752	3.4763	0.000508	0.075517	Insyn1
20384	-0.19317	-3.4459	0.000569	0.080226	Srsf5
27981	-0.21925	-4.3185	1.57E-05	0.009911	Rsrp1
432731	-0.22012	-3.4199	0.000626	0.08256	Zscan26
235134	-0.25629	-3.6131	0.000303	0.05258	Nfrkb
83701	-0.26019	-3.4845	0.000493	0.074678	Srrt
66826	-0.26595	-3.4675	0.000525	0.076846	Taz
15547	-0.26596	-3.3648	0.000766	0.092422	Trmt2a
224907	-0.26621	-3.8758	0.000106	0.030263	Dus3l
110809	-0.27018	-3.8345	0.000126	0.034005	Srsf1
217935	-0.27511	-3.8032	0.000143	0.036071	Wdr60
70430	-0.28065	-3.438	0.000586	0.080226	Tbce
51796	-0.28773	-3.8911	9.98E-05	0.02919	Srrm1
71742	-0.29053	-3.4029	0.000667	0.084745	Ulk3
20908	-0.29663	-3.3564	0.00079	0.093693	Stx3
16400	-0.29845	-3.6614	0.000251	0.049601	Itga3
382793	-0.29905	-3.6382	0.000275	0.051888	Mtx3
55935	-0.30147	-3.4382	0.000586	0.080226	Fnbp4
101351	-0.30241	-3.749	0.000178	0.041207	Eogt
269401	-0.30696	-3.3661	0.000762	0.092422	Zfp512b
19303	-0.31216	-3.799	0.000145	0.036071	Pxn
243547	-0.31655	-3.6128	0.000303	0.05258	Grip2
22221	-0.31802	-4.0931	4.26E-05	0.016228	Ubp1
224902	-0.32086	-3.5196	0.000432	0.068884	Safb2
319758	-0.32947	-3.4433	0.000575	0.080226	Rfx7
52535	-0.33413	-3.3564	0.00079	0.093693	Mettl17
233833	-0.33697	-4.2249	2.39E-05	0.011259	Tnrc6a
319670	-0.33711	-3.6422	0.00027	0.051537	Eml5
109135	-0.34078	-3.6265	0.000287	0.052033	Plekha5
103135	-0.34895	-3.4249	0.000615	0.081834	Pan2
57330	-0.35021	-3.9688	7.22E-05	0.023375	Gigyf1
53609	-0.35302	-3.9149	9.05E-05	0.027052	Clasrp
13543	-0.35974	-4.1071	4.01E-05	0.01556	Dvl2
237943	-0.36061	-3.4536	0.000553	0.079821	Gpatch8

© 2025. Thadathil N et al. Published online at http://www.aginganddisease.org/EN/10.14336/AD.2025.0805

56399	-0.36135	-5.5365	3.09E-08	0.000132	Akap8
69440	-0.36187	-4.2058	2.60E-05	0.011338	Dennd6b
74480	-0.36998	-3.4739	0.000513	0.075542	Samd4
231003	-0.37028	-5.8259	5.68E-09	4.04E-05	Klhl17
26412	-0.37127	-4.4425	8.89E-06	0.007154	Map4k2
234686	-0.37141	-3.6653	0.000247	0.049601	Fhod1
54194	-0.37936	-4.2611	2.03E-05	0.010343	Akap8l
14789	-0.37992	-3.6153	0.0003	0.05258	P3h3
212728	-0.38248	-3.683	0.000231	0.047335	Tarbp1
227644	-0.38345	-4.2994	1.71E-05	0.009911	Snapc4
103537	-0.38544	-3.6098	0.000306	0.052779	Mbtd1
72826	-0.38837	-3.424	0.000617	0.081834	Fam76b
72039	-0.39182	-3.7086	0.000208	0.044944	Mccc1
19732	-0.39312	-4.2755	1.91E-05	0.010183	Rgl2
26920	-0.39608	-3.34	0.000838	0.098789	Cntrl
213054	-0.39784	-3.3739	0.000741	0.09096	Gabpb2
76491	-0.3989	-4.2668	1.98E-05	0.010326	Abhd14b
319899	-0.40345	-3.3904	0.000698	0.088182	Dock6
235323	-0.40614	-3.9958	6.45E-05	0.021859	Usp28
18763	-0.40767	-3.4275	0.000609	0.081834	Pkd1
11781	-0.41096	-3.6706	0.000242	0.049214	Ap4m1
20592	-0.41192	-3.4866	0.000489	0.074604	Kdm5d
58988	-0.41375	-3.725	0.000195	0.043449	Rps6kb2
381644	-0.41387	-3.4946	0.000475	0.073469	Cep135
29856	-0.41609	-3.3719	0.000746	0.091089	Smtn
16801	-0.41868	-3.5686	0.000359	0.058958	Arhgef1
12815	-0.41938	-3.8308	0.000128	0.034095	Col11a2
207777	-0.41955	-3.6924	0.000222	0.046511	Tspoap1
66435	-0.42112	-3.5348	0.000408	0.066014	Uggt2
192176	-0.42343	-4.0681	4.74E-05	0.017451	Flna
232560	-0.42432	-3.4403	0.000581	0.080226	Caprin2
319317	-0.42902	-3.9255	8.65E-05	0.026976	Snhg11
56878	-0.43681	-3.6277	0.000286	0.052033	Rbms1
59024	-0.44045	-4.2906	1.78E-05	0.009911	Med12
22658	-0.44415	-3.7693	0.000164	0.03885	Pcgf2
218294	-0.44652	-4.4905	7.11E-06	0.00607	Cdc14b
230676	-0.44661	-3.6265	0.000287	0.052033	Szt2
224697	-0.44687	-3.9129	9.12E-05	0.027052	Adamts10
217366	-0.4507	-4.3562	1.32E-05	0.009578	Lrrc45
224814	-0.46026	-4.0475	5.18E-05	0.018738	Abcc10
68074	-0.4614	-3.6263	0.000288	0.052033	A930013F10Rik
70615	-0.46651	-4.2275	2.36E-05	0.011259	Ankrd24
110962	-0.47144	-4.6486	3.34E-06	0.003568	Mbd6
12395	-0.47703	-3.4398	0.000582	0.080226	Runx1t1
103836	-0.47867	-4.2891	1.79E-05	0.009911	Zfp692
75956	-0.51193	-4.029	5.60E-05	0.019715	Srrm2
208266	-0.51298	-3.7255	0.000195	0.043449	Dot1l
230872	-0.53182	-4.3037	1.68E-05	0.009911	Crocc
232798	-0.53811	-4.7996	1.59E-06	0.002122	Leng8
107934	-0.5483	-3.9235	8.73E-05	0.026976	Celsr3
329584	-0.55855	-4.1379	3.51E-05	0.014123	Slc2a4rg-ps
103213	-0.56038	-4.2216	2.43E-05	0.011259	Traf3ip2
17263	-0.56337	-4.5032	6.70E-06	0.005957	Meg3

© 2025. Thadathil N et al. Published online at http://www.aginganddisease.org/EN/10.14336/AD.2025.0805

1.01E+08	-0.56967	-3.8218	0.000132	0.034557	2810029C07Rik
50782	-0.57305	-5.1962	2.03E-07	0.000395	Rgs11
101883	-0.58123	-3.6167	0.000298	0.05258	Igflr1
1.01E+08	-0.60453	-3.8013	0.000144	0.036071	Mir5125
1E+08	-0.69971	-5.3282	9.92E-08	0.000265	D030047H15Rik
17540	-0.72862	-3.8814	0.000104	0.029972	Mrvi1
11443	-0.75012	-4.3821	1.18E-05	0.008965	Chrnb1
69750	-0.77773	-3.4341	0.000594	0.080852	2410021H03Rik
237611	-0.82441	-4.175	2.98E-05	0.012727	Stac3
20350	-0.83921	-4.6512	3.30E-06	0.003568	Sema3f
66961	-0.85563	-4.077	4.56E-05	0.017088	Neat1
68144	-0.86075	-4.5256	6.02E-06	0.005592	5031426D15Rik
216961	-0.86886	-3.8003	0.000145	0.036071	Coro6
26365	-0.88414	-4.2504	2.13E-05	0.010599	Ceacam1
381853	-0.89862	-4.2871	1.81E-05	0.009911	Gipr
27062	-0.90078	-6.2841	3.30E-10	3.52E-06	Cadps
408192	-0.90279	-3.4986	0.000468	0.072904	Gm9839
106766	-0.9382	-3.7761	0.000159	0.03822	Stap2
1E+08	-0.93841	-4.8466	1.26E-06	0.001788	E030047D23Rik
333088	-1.0006	-3.3341	0.000856	0.099332	Kcp
619309	-1.0285	-4.9483	7.49E-07	0.001142	Muc3a
320749	-1.0431	-3.9203	8.84E-05	0.026976	D630041G03Rik
20514	-1.0921	-4.5973	4.28E-06	0.004352	Slc1a5
408191	-1.1615	-4.2964	1.74E-05	0.009911	Gm5415
353499	-1.2365	-7.1137	1.13E-12	2.41E-08	Tmc4
1E+08	-1.3351	-3.3836	0.000715	0.089145	Gm4583
232790	-1.4026	-4.708	2.50E-06	0.002968	Oscar
243864	-1.4349	-4.3526	1.35E-05	0.009578	Mill2
330403	-1.4448	-3.8214	0.000133	0.034557	Gm9946
1.01E+08	-1.4918	-5.4492	5.06E-08	0.000175	Gm20371
69732	-1.7851	-3.6879	0.000226	0.04687	2410018L13Rik
67425	-1.9818	-5.2722	1.35E-07	0.000304	Eps8l1
328783	-2.2825	-3.4059	0.000659	0.084324	Mslnl

Supplementary Table 2. KEGG pathway enrichment analysis of differentially expressed genes in nMlkl-KI mice. Gene Set Enrichment Analysis (GSEA) was performed to identify significantly

© 2025. Thadathil N et al. Published online at http://www.aginganddisease.org/EN/10.14336/AD.2025.0805

enriched KEGG pathways in 12-months old Slick H Cre-Rosa Mlkl mice. The 12 pathways significantly (P-values ranging from 0.02 to 2 x 10^{-15}) altered in the cortex of nMlkl-KI mice are listed. The total genes in each pathway as well as the number of hits (genes within the pathway that contribute to the enrichment) in the pathway that are altered are shown. Enrichment Score (ES) measures how strongly a predefined gene set is enriched at the top or bottom of a ranked gene list.

**Pathway**	**Total Genes**	**Hits**	**Enrichment Score**	**P-Value**
Ribosome	175	135	0.698	2e-15
Parkinson’s disease	144	144	0.477	9e-05
Oxidative phosphorylation	134	131	0.494	0.0001
Neuroactive ligand-receptor interaction	348	316	-0.422	0.0005
Proteosome	46	44	0.618	0.0008
Huntington's disease	194	192	0.386	0.004
Chemokine signaling pathway	200	184	0.378	0.007
Non-alcoholic fatty liver disease (NAFLD)	151	150	0.389	0.008
Olfactory transduction	1134	185	-0.410	0.018
Amoebiasis	175	173	-0.362	0.022
Alzheimer’s disease	175	173	0.362	0.022
TNF Signaling	110	107	0.390	0.026

© 2025. Thadathil N et al. Published online at http://www.aginganddisease.org/EN/10.14336/AD.2025.0805
